# *PALB2*, *CHEK2* and *ATM* rare variants and cancer risk: data from COGS

**DOI:** 10.1136/jmedgenet-2016-103839

**Published:** 2016-09-05

**Authors:** Melissa C Southey, David E Goldgar, Robert Winqvist, Katri Pylkäs, Fergus Couch, Marc Tischkowitz, William D Foulkes, Joe Dennis, Kyriaki Michailidou, Elizabeth J van Rensburg, Tuomas Heikkinen, Heli Nevanlinna, John L Hopper, Thilo Dörk, Kathleen BM Claes, Jorge Reis-Filho, Zhi Ling Teo, Paolo Radice, Irene Catucci, Paolo Peterlongo, Helen Tsimiklis, Fabrice A Odefrey, James G Dowty, Marjanka K Schmidt, Annegien Broeks, Frans B Hogervorst, Senno Verhoef, Jane Carpenter, Christine Clarke, Rodney J Scott, Peter A Fasching, Lothar Haeberle, Arif B Ekici, Matthias W Beckmann, Julian Peto, Isabel dos-Santos-Silva, Olivia Fletcher, Nichola Johnson, Manjeet K Bolla, Elinor J Sawyer, Ian Tomlinson, Michael J Kerin, Nicola Miller, Federik Marme, Barbara Burwinkel, Rongxi Yang, Pascal Guénel, Thérèse Truong, Florence Menegaux, Marie Sanchez, Stig Bojesen, Sune F Nielsen, Henrik Flyger, Javier Benitez, M Pilar Zamora, Jose Ignacio Arias Perez, Primitiva Menéndez, Hoda Anton-Culver, Susan Neuhausen, Argyrios Ziogas, Christina A Clarke, Hermann Brenner, Volker Arndt, Christa Stegmaier, Hiltrud Brauch, Thomas Brüning, Yon-Dschun Ko, Taru A Muranen, Kristiina Aittomäki, Carl Blomqvist, Natalia V Bogdanova, Natalia N Antonenkova, Annika Lindblom, Sara Margolin, Arto Mannermaa, Vesa Kataja, Veli-Matti Kosma, Jaana M Hartikainen, Amanda B Spurdle, kConFab Investigators, Australian Ovarian Cancer Study Group, Els Wauters, Dominiek Smeets, Benoit Beuselinck, Giuseppe Floris, Jenny Chang-Claude, Anja Rudolph, Petra Seibold, Dieter Flesch-Janys, Janet E Olson, Celine Vachon, Vernon S Pankratz, Catriona McLean, Christopher A Haiman, Brian E Henderson, Fredrick Schumacher, Loic Le Marchand, Vessela Kristensen, Grethe Grenaker Alnæs, Wei Zheng, David J Hunter, Sara Lindstrom, Susan E Hankinson, Peter Kraft, Irene Andrulis, Julia A Knight, Gord Glendon, Anna Marie Mulligan, Arja Jukkola-Vuorinen, Mervi Grip, Saila Kauppila, Peter Devilee, Robert A E M Tollenaar, Caroline Seynaeve, Antoinette Hollestelle, Montserrat Garcia-Closas, Jonine Figueroa, Stephen J Chanock, Jolanta Lissowska, Kamila Czene, Hatef Darabi, Mikael Eriksson, Diana M Eccles, Sajjad Rafiq, William J Tapper, Sue M Gerty, Maartje J Hooning, John W M Martens, J Margriet Collée, Madeleine Tilanus-Linthorst, Per Hall, Jingmei Li, Judith S Brand, Keith Humphreys, Angela Cox, Malcolm W R Reed, Craig Luccarini, Caroline Baynes, Alison M Dunning, Ute Hamann, Diana Torres, Hans Ulrich Ulmer, Thomas Rüdiger, Anna Jakubowska, Jan Lubinski, Katarzyna Jaworska, Katarzyna Durda, Susan Slager, Amanda E Toland, Christine B Ambrosone, Drakoulis Yannoukakos, Anthony Swerdlow, Alan Ashworth, Nick Orr, Michael Jones, Anna González-Neira, Guillermo Pita, M Rosario Alonso, Nuria Álvarez, Daniel Herrero, Daniel C Tessier, Daniel Vincent, Francois Bacot, Jacques Simard, Martine Dumont, Penny Soucy, Rosalind Eeles, Kenneth Muir, Fredrik Wiklund, Henrik Gronberg, Johanna Schleutker, Børge G Nordestgaard, Maren Weischer, Ruth C Travis, David Neal, Jenny L Donovan, Freddie C Hamdy, Kay-Tee Khaw, Janet L Stanford, William J Blot, Stephen Thibodeau, Daniel J Schaid, Joseph L Kelley, Christiane Maier, Adam S Kibel, Cezary Cybulski, Lisa Cannon-Albright, Katja Butterbach, Jong Park, Radka Kaneva, Jyotsna Batra, Manuel R Teixeira, Zsofia Kote-Jarai, Ali Amin Al Olama, Sara Benlloch, Stefan P Renner, Arndt Hartmann, Alexander Hein, Matthias Ruebner, Diether Lambrechts, Els Van Nieuwenhuysen, Ignace Vergote, Sandrina Lambretchs, Jennifer A Doherty, Mary Anne Rossing, Stefan Nickels, Ursula Eilber, Shan Wang-Gohrke, Kunle Odunsi, Lara E Sucheston-Campbell, Grace Friel, Galina Lurie, Jeffrey L Killeen, Lynne R Wilkens, Marc T Goodman, Ingo Runnebaum, Peter A Hillemanns, Liisa M Pelttari, Ralf Butzow, Francesmary Modugno, Robert P Edwards, Roberta B Ness, Kirsten B Moysich, Andreas du Bois, Florian Heitz, Philipp Harter, Stefan Kommoss, Beth Y Karlan, Christine Walsh, Jenny Lester, Allan Jensen, Susanne Krüger Kjaer, Estrid Høgdall, Bernard Peissel, Bernardo Bonanni, Loris Bernard, Ellen L Goode, Brooke L Fridley, Robert A Vierkant, Julie M Cunningham, Melissa C Larson, Zachary C Fogarty, Kimberly R Kalli, Dong Liang, Karen H Lu, Michelle A T Hildebrandt, Xifeng Wu, Douglas A Levine, Fanny Dao, Maria Bisogna, Andrew Berchuck, Edwin S Iversen, Jeffrey R Marks, Lucy Akushevich, Daniel W Cramer, Joellen Schildkraut, Kathryn L Terry, Elizabeth M Poole, Meir Stampfer, Shelley S Tworoger, Elisa V Bandera, Irene Orlow, Sara H Olson, Line Bjorge, Helga B Salvesen, Anne M van Altena, Katja K H Aben, Lambertus A Kiemeney, Leon F A G Massuger, Tanja Pejovic, Yukie Bean, Angela Brooks-Wilson, Linda E Kelemen, Linda S Cook, Nhu D Le, Bohdan Górski, Jacek Gronwald, Janusz Menkiszak, Claus K Høgdall, Lene Lundvall, Lotte Nedergaard, Svend Aage Engelholm, Ed Dicks, Jonathan Tyrer, Ian Campbell, Iain McNeish, James Paul, Nadeem Siddiqui, Rosalind Glasspool, Alice S Whittemore, Joseph H Rothstein, Valerie McGuire, Weiva Sieh, Hui Cai, Xiao-Ou Shu, Rachel T Teten, Rebecca Sutphen, John R McLaughlin, Steven A Narod, Catherine M Phelan, Alvaro N Monteiro, David Fenstermacher, Hui-Yi Lin, Jennifer B Permuth, Thomas A Sellers, Y Ann Chen, Ya-Yu Tsai, Zhihua Chen, Aleksandra Gentry-Maharaj, Simon A Gayther, Susan J Ramus, Usha Menon, Anna H Wu, Celeste L Pearce, David Van Den Berg, Malcolm C Pike, Agnieszka Dansonka-Mieszkowska, Joanna Plisiecka-Halasa, Joanna Moes-Sosnowska, Jolanta Kupryjanczyk, Paul DP Pharoah, Honglin Song, Ingrid Winship, Georgia Chenevix-Trench, Graham G Giles, Sean V Tavtigian, Doug F Easton, Roger L Milne

**Affiliations:** 1 Genetic Epidemiology Laboratory, Department of Pathology, The University of Melbourne, Melbourne, Australia; 2 Huntsman Cancer Institute, Salt Lake City, UT, USA; 3 Laboratory of Cancer Genetics and Tumor Biology, Cancer and Translational Medicine Research Unit and Biocenter Oulu, University of Oulu, Nordlab Oulu, Oulu, Finland; 4 Department of Laboratory Medicine and Pathology, Mayo Clinic, Rochester, MN, USA; 5 Department of Medical Genetics and National Institute for Health Research Cambridge Biomedical Research Centre, University of Cambridge, and the Department of Clinical Genetics, East Anglian Regional Genetics Service, Addenbrooke's Hospital; 6 Program in Cancer Genetics, Department of Human Genetics and Oncology, Lady Davis Institute, and Research Institute, McGill University Health Centre, McGill University, Montreal, Canada,; 7 Centre for Cancer Genetic Epidemiology, Department of Public Health and Primary Care, University of Cambridge, Strangeways Laboratory, Worts Causeway, Cambridge, UK; 8 Department of Genetics, University of Pretoria, South Africa; 9 Department of Obstetrics and Gynecology, University of Helsinki and Helsinki University Central Hospital, Helsinki, Finland; 10 Centre for Epidemiology and Biostatistics, School of Population and Global Health, The University of Melbourne, Melbourne, Australia,; 11 Gynaecology Research Unit, Hannover Medical School, Hannover, Germany; 12 Center for Medical Genetics, Ghent University Hospital, De Pintelaan 185, 9000 Ghent, Belgium,; 13 Department of Pathology and Human Oncology and Pathogenesis Program, Memorial Sloan-Kettering Cancer Center, New York, New York, USA; 14 Unit of Molecular Bases of Genetic Risk and Genetic Testing, Department of Preventive and Predictive Medicine, Fondazione IRCCS Istituto Nazionale dei Tumori (INT), Milan, Italy; 15 IFOM, the FIRC Institute of Molecular Oncology, Milan, Italy; 16 Netherlands Cancer Institute, Antoni van Leeuwenhoek hospital, Amsterdam, The Netherlands; 17 Australian Breast Cancer Tissue Bank, University of Sydney at the Westmead Institute for Medical Research, NSW, Australia; 18 Centre for Cancer Research, University of Sydney at the Westmead Institute for Medical Research, NSW, Australia; 19 Division of Molecular Medicine, Pathology North, Newcastle and University of Newcastle, NSW, Australia; 20 University Breast Center Franconia, Department of Gynecology and Obstetrics, University Hospital Erlangen, Friedrich-Alexander University Erlangen-Nuremberg, Comprehensive Cancer Center Erlangen-EMN, Erlangen, Germany; 21 David Geffen School of Medicine, Department of Medicine Division of Hematology and Oncology, University of California at Los Angeles, CA, USA; 22 Unit of Biostatistics, Department of Gynecology and Obstetrics, University Hospital Erlangen, Friedrich-Alexander University Erlangen-Nuremberg, Erlangen, Germany; 23 Institute of Human Genetics, University Hospital Erlangen, Friedrich Alexander University Erlangen-Nuremberg, Erlangen, Germany; 24 Non-communicable Disease Epidemiology Department, London School of Hygiene and Tropical Medicine, London, UK; 25 Breakthrough Breast Cancer Research Centre, The Institute of Cancer Research, London, UK; 26 Division of Cancer Studies, NIHR Comprehensive Biomedical Research Centre, Guy's & St. Thomas' NHS Foundation Trust in partnership with King's College London, London, UK; 27 Wellcome Trust Centre for Human Genetics and Oxford Biomedical Research Centre, University of Oxford, UK and Oxford NIHR Biomedical Research Centre, Headington, OX3 7LE; 28 Surgery, Lambe Institute for Translational Science, NUIGalway, University Hospital Galway, Galway, Ireland; 29 Department of Obstetrics and Gynecology, University of Heidelberg, Heidelberg, Germany; 30 National Center for Tumor Diseases, University of Heidelberg, Heidelberg, Germany; 31 Molecular Epidemiology Group, German Cancer Research Center (DKFZ), Heidelberg, Germany; 32 Inserm (National Institute of Health and Medical Research), CESP (Center for Research in Epidemiology and Population Health), U1018, Environmental Epidemiology of Cancer, Villejuif, France; 33 University Paris-Sud, UMRS 1018, Villejuif, France; 34 Copenhagen General Population Study, Herlev Hospital, Copenhagen University Hospital, University of Copenhagen, Copenhagen, Denmark; 35 Department of Clinical Biochemistry, Herlev Hospital, Copenhagen University Hospital, University of Copenhagen, Copenhagen, Denmark; 36 Department of Breast Surgery, Herlev Hospital, Copenhagen University Hospital, Copenhagen, Denmark; 37 Human Genetics Group, Human Cancer Genetics Program, Spanish National Cancer Research Centre (CNIO), Madrid, Spain; 38 Centro de Investigación en Red de Enfermedades Raras (CIBERER), Valencia, Spain; 39 Servicio de Oncología Médica, Hospital Universitario La Paz, Madrid, Spain; 40 Servicio de Cirugía General y Especialidades, Hospital Monte Naranco, Oviedo, Spain; 41 Servicio de Anatomía Patológica, Hospital Monte Naranco, Oviedo, Spain; 42 Department of Epidemiology, University of California Irvine, Irvine, California, USA; 43 Beckman Research Institute of City of Hope, Duarte, California, USA; 44 Department of Epidemiology, University of California Irvine, Irvine, California, USA; 45 Cancer Prevention Institute of California, Fremont, California, USA; 46 Division of Clinical Epidemiology and Aging Research, German Cancer Research Center (DKFZ), Heidelberg, Germany; 47 Division of Preventive Oncology, German Cancer Research Center (DKFZ), Heidelberg, Germany; 48 German Cancer Consortium (DKTK), German Cancer Research Center (DKFZ), Heidelberg, Germany; 49 Saarland Cancer Registry, Saarbrücken, Germany; 50 Dr. Margarete Fischer-Bosch-Institute of Clinical Pharmacology, Stuttgart; 51 University of Tübingen, Tübingen, Germany; 52 Institute for Prevention and Occupational Medicine of the German Social Accident Insurance, Institute of the Ruhr University, Bochum (IPA), Germany; 53 Department of Internal Medicine, Evangelische Kliniken Bonn gGmbH, Johanniter Krankenhaus, Bonn, Germany; 54 Department of Obstetrics and Gynecology, University of Helsinki and Helsinki University Central Hospital, Helsinki, Finland; 55 Department of Clinical Genetics, Helsinki University Central Hospital, Helsinki, Finland; 56 Department of Oncology, Helsinki University Central Hospital, Helsinki, Finland; 57 Department of Radiation Oncology, Hannover Medical School, Hannover, Germany; 58 N.N. Alexandrov Research Institute of Oncology and Medical Radiology, Minsk, Belarus; 59 Department of Molecular Medicine and Surgery, Karolinska Institutet, Stockholm, Sweden; 60 Department of Oncology – Pathology, Karolinska Institutet, Stockholm, Sweden; 61 School of Medicine, Institute of Clinical Medicine, Pathology and Forensic Medicine, and Cancer Center of Eastern Finland, University of Eastern Finland, Kuopio, Finland; 62 Imaging Center, Department of Clinical Pathology, Kuopio University Hospital, Kuopio, Finland; 63 School of Medicine, Institute of Clinical Medicine, Oncology, University of Eastern Finland, Kuopio, Finland; 64 Biocenter Kuopio, Cancer Center of Eastern Finland, Kuopio University Hospital, Kuopio, Finland; 65 QIMR Berghofer Medical Research Institute, Brisbane, Australia; 66 Research Department, Peter MacCallum Cancer Centre and The Sir Peter MacCallum Department of Oncology, University of Melbourne, Victoria, Australia; 67 Vesalius Research Center (VRC), VIB, Leuven, Belgium; 68 Laboratory for Translational Genetics, Department of Oncology, University of Leuven, Leuven, Belgium; 69 University Hospital Gasthuisberg, Leuven, Belgium; 70 Division of Cancer Epidemiology, German Cancer Research Center (DKFZ), Heidelberg, Germany; 71 Department of Cancer Epidemiology/Clinical Cancer Registry and Institute for Medical Biometrics and Epidemiology, University Clinic Hamburg-Eppendorf, Hamburg, Germany; 72 Department of Health Sciences Research, Mayo Clinic, Rochester, MN, USA; 73 Anatomical Pathology, The Alfred Hospital, Melbourne, Australia; 74 Department of Preventive Medicine, Keck School of Medicine, University of Southern California, Los Angeles, CA, USA; 75 Epidemiology Program, Cancer Research Center, University of Hawaii, Honolulu, HI, USA; 76 Department of Genetics, Institute for Cancer Research, Oslo University Hospital, Radiumhospitalet, Oslo, Norway; 77 Faculty of Medicine (Faculty Division Ahus), University of Oslo (UiO), Norway; 78 Division of Epidemiology, Department of Medicine, Vanderbilt Epidemiology Center, Vanderbilt-Ingram Cancer Center, Vanderbilt University School of Medicine, Nashville, TN, USA; 79 Program in Molecular and Genetic Epidemiology, Harvard School of Public Health, Boston, MA, USA; 80 Department of Epidemiology, Harvard School of Public Health, Boston, MA, USA; 81 Channing Laboratory, Department of Medicine, Brigham and Women's Hospital and Harvard Medical School, Boston, MA, USA; 82 Ontario Cancer Genetics Network, Lunenfeld-Tanenbaum Research Institute of Mount Sinai Hospital, Toronto, Ontario, Canada; 83 Department of Molecular Genetics, University of Toronto, Toronto, Ontario, Canada; 84 Prosserman Centre for Health Research, Lunenfeld-Tanenbaum Research Institute of Mount Sinai Hospital, Toronto, Ontario, Canada; 85 Division of Epidemiology, Dalla Lana School of Public Health, University of Toronto, Toronto, Ontario, Canada; 86 Department of Laboratory Medicine and Pathobiology, University of Toronto, Toronto, ON, Canada; 87 Laboratory Medicine Program, University Health Network, Toronto, Ontario; Department of Laboratory Medicine and Pathobiology, University of Toronto, Toronto, ON, Canada; 88 Department of Oncology, Oulu University Hospital, University of Oulu, Oulu, Finland; 89 Department of Surgery, Oulu University Hospital, University of Oulu, Oulu, Finland; 90 Department of Pathology, Oulu University Hospital, University of Oulu, Oulu, Finland; 91 Department of Surgical Oncology, Leiden University Medical Center, 2300 RC Leiden, The Netherlands; 92 Family Cancer Clinic, Department of Medical Oncology, Erasmus MC-Daniel den Hoed Cancer Centre, Rotterdam, The Netherlands; 93 The Breast Cancer Now Toby Robins Research Centre, The Institute of Cancer Research, London, SW3 6JB, UK; 94 Division of Cancer Epidemiology and Genetics, National Cancer Institute, Rockville, Maryland, USA; 95 Department of Cancer Epidemiology and Prevention, M. Sklodowska-Curie Memorial Cancer Center & Institute of Oncology, Warsaw, Poland; 96 Department of Medical Epidemiology and Biostatistics, Karolinska Institutet, Stockholm 17177, Sweden; 97 Faculty of Medicine, University of Southampton (UoS), Southampton UK; 98 Department of Medical Oncology, Family Cancer Clinic, Erasmus MC Cancer Institute, Rotterdam, The Netherlands; 99 Department of Clinical Genetics, Family Cancer Clinic, Erasmus University Medical Center, Rotterdam, The Netherlands; 100 Department of Surgical Oncology, Family Cancer Clinic, Erasmus University Medical Center, Rotterdam, The Netherlands; 101 Department of Medical Epidemiology and Biostatistics, Karolinska Institutet, Stockholm 17177, Sweden; 102 Human Genetics Division, Genome Institute of Singapore, Singapore 138672, Singapore; 103 Sheffield Cancer Research, Department of Oncology, University of Sheffield, Sheffield, UK; 104 Centre for Cancer Genetic Epidemiology, Department of Oncology, University of Cambridge, Cambridge, UK; 105 Molecular Genetics of Breast Cancer, German Cancer Research Center (DKFZ), Heidelberg, Germany; 106 Institute of Human Genetics, Pontificia Universidad Javeriana, Bogota, Colombia; 107 Frauenklinik der Stadtklinik Baden-Baden, Baden-Baden, Germany; 108 Institute of Pathology, Städtisches Klinikum Karlsruhe, Karlsruhe, Germany; 109 Department of Genetics and Pathology, Pomeranian Medical University, Szczecin, Poland; 110 Postgraduate School of Molecular Medicine, Warsaw Medical University, Warsaw, Poland; 111 Department of Molecular Virology, Immunology and Medical Genetics, Comprehensive Cancer Center, The Ohio State University, Columbus, OH, USA; 112 Roswell Park Cancer Institute, Buffalo, New York, USA; 113 Molecular Diagnostics Laboratory, IRRP, National Centre for Scientific Research "Demokritos", Aghia Paraskevi Attikis, Athens, Greece; 114 Division of Genetics and Epidemiology, Institute of Cancer Research, London, UK; 115 Division of Breast Cancer Research, Institute of Cancer Research, London, UK; 116 Centre d'innovation Genome Quebec et University McGill Montreal Quebec, Canada; 117 McGill University, Montreal, Quebec, Canada; 118 Cancer Genomics Laboratory, Centre Hospitalier Universitaire de Quebec Research Center. Laval University, Quebec, Canada; 119 The Institute of Cancer Research, London, SM2 5NG, UK; 120 Royal Marsden NHS Foundation Trust, Fulham, London, SW3 6JJ, UK; 121 University of Warwick, Coventry, UK; 122 Department of Medical Epidemiology and Biostatistics, Karolinska Institute, Stockholm, Sweden; 123 Department of Medical Biochemistry and Genetics, University of Turku, and Tyks Microbiology and Genetics, Department of Medical Genetics, Turku University Hospital, Turku, Finland; 124 Institute of Biomedical Technology/BioMediTech, University of Tampere, Tampere, Finland; 125 Department of Clinical Biochemistry, Herlev Hospital, Copenhagen University Hospital, Herlev Ringvej 75, DK-2730 Herlev, Denmark; 126 Department of Human Genetics University of Utah, Salt Lake City, UT, USA and Department of Clinical Biochemistry, Herlev Hospital, Copenhagen University Hospital, University of Copenhagen, Copenhagen, Denmark; 127 Cancer Epidemiology Unit, Nuffield Department of Population Health, University of Oxford, Oxford, UK; 128 Surgical Oncology (Uro-Oncology: S4), University of Cambridge, Box 279, Addenbrooke's Hospital, Hills Road, Cambridge, UK and Cancer Research UK Cambridge Research Institute, Li Ka Shing Centre, Cambridge, UK; 129 Professor of Social Medicine, University of Bristol, Canynge Hall, 39 Whatley Road, Bristol BS8 2PS; 130 Nuffield Department of Surgical Sciences, Old Road Campus Research Building (off Roosevelt Drive), University of Oxford, Headington, Oxford, OX3 7DQ; 131 Cambridge Institute of Public Health, University of Cambridge, Forvie Site, Robinson Way, Cambridge CB2 0SR; 132 Division of Public Health Sciences, Fred Hutchinson Cancer Research Center, Seattle, Washington, USA; 133 Department of Epidemiology, School of Public Health, University of Washington, Seattle, Washington, USA; 134 International Epidemiology Institute, 1455 Research Blvd., Suite 550, Rockville, MD 20850; 135 Department of Obstetrics, Gynecology and Reproductive Sciences, University of Pittsburgh School of Medicine, Pittsburgh, PA, USA; 136 Department of Urology, University Hospital Ulm, Germany; 137 Institute of Human Genetics University Hospital Ulm, Germany; 138 Brigham and Women's Hospital/Dana-Farber Cancer Institute, 45 Francis Street- ASB II-3, Boston, MA 02115; 139 Washington University, St Louis, Missouri; 140 International Hereditary Cancer Center, Department of Genetics and Pathology, Pomeranian Medical University, Szczecin, Poland; 141 Division of Genetic Epidemiology, Department of Medicine, University of Utah School of Medicine; 142 Division of Cancer Prevention and Control, H. Lee Moffitt Cancer Center, 12902 Magnolia Dr., Tampa, Florida, USA; 143 Molecular Medicine Center and Department of Medical Chemistry and Biochemistry, Medical University – Sofia, 2 Zdrave St, 1431, Sofia, Bulgaria; 144 Australian Prostate Cancer Research Centre-Qld, Institute of Health and Biomedical Innovation and Schools of Life Science and Public Health, Queensland University of Technology, Brisbane, Australia; 145 Department of Genetics, Portuguese Oncology Institute, Porto, Portugal and Biomedical Sciences Institute (ICBAS), Porto University, Porto, Portugal; 146 University Hospital Erlangen, Department of Gynecology and Obstetrics, Friedrich-Alexander-University Erlangen-Nuremberg, Comprehensive Cancer Center Erlangen-EMN, Universitaetsstrasse 21-23, 91054 Erlangen, Germany; 147 University Hospital Erlangen, Institute of Pathology, Friedrich-Alexander-University Erlangen-Nuremberg, Comprehensive Cancer Center Erlangen-EMN, Universitaetsstrasse 21-23, 91054 Erlangen, German; 148 Vesalius Research Center, VIB, Leuven, Belgium; 149 Laboratory for Translational Genetics, Department of Oncology, University of Leuven, Belgium; 150 Department of Epidemiology, The Geisel School of Medicine at Dartmouth, Lebanon, NH, USA; 151 Department of Epidemiology, The Geisel School of Medicine at Dartmouth, Hannover, NH, USA; 152 Program in Epidemiology, Division of Public Health Sciences, Fred Hutchinson Cancer Research Center, Seattle, WA, USA; 153 Department of Epidemiology, University of Washington, Seattle, WA, USA; 154 German Cancer Research Center, Division of Cancer Epidemiology, Heidelberg, Germany; 155 Department of Obstetrics and Gynecology, University of Ulm, Ulm, Germany; 156 Department of Gynecological Oncology, Roswell Park Cancer Institute, Buffalo, NY; 157 Cancer Epidemiology Program, University of Hawaii Cancer Center, Hawaii, USA; 158 Department of Pathology, Kapiolani Medical Center for Women and Children, John A. Burns School of Medicine, University of Hawaii, Honolulu, Hawaii 96826, USA; 159 Cancer Prevention and Control, Samuel Oschin Comprehensive Cancer Institute, Cedars-Sinai Medical Center, Los Angeles, California, USA; 160 Community and Population Health Research Institute, Department of Biomedical Sciences, Cedars-Sinai Medical Center, Los Angeles, California, USA; 161 Department of Gynecology and Obstetrics, Friedrich Schiller University, Jena University Hospital, Jena, Germany; 162 Clinics of Obstetrics and Gynaecology, Hannover Medical School, Hannover, Germany; 163 Department of Pathology, Helsinki University Central Hospital, Helsinki, 00029 HUS, Finland; 164 University of Pittsburgh Department of Obstetrics, Gynecology and Reproductive Sciences and Ovarian Cancer Center of Excellence Pittsburgh PA USA; 165 University of Pittsburgh Department of Epidemiology, University of Pittsburgh Graduate School of Public Health and Womens Cancer Research Program, Magee-Womens Research Institute and University of Pittsburgh Cancer Institute Pittsburgh PA USA; 166 The University of Texas School of Public Health, Houston, TX, USA; 167 Department of Cancer Prevention and Control, Roswell Park Cancer Institute, Buffalo, NY; 168 Department of Gynecology and Gynecologic Oncology, Kliniken Essen-Mitte/ Evang. Huyssens-Stiftung/ Knappschaft GmbH, Essen, Germany; 169 Department of Gynecology and Gynecologic Oncology, Dr. Horst Schmidt Kliniken Wiesbaden, Wiesbaden, Germany; 170 Tuebingen University Hospital, Department of Women's Health, Tuebingen, Germany; 171 Women's Cancer Program at the Samuel Oschin Comprehensive Cancer Institute, Cedars-Sinai Medical Center, Los Angeles, California; 172 Department of Virus, Lifestyle and Genes, Danish Cancer Society Research Center, Copenhagen, Denmark; 173 Department of Obstetrics and Gynecology, Rigshospitalet, Copenhagen, Denmark; 174 Molecular Unit, Department of Pathology, Herlev Hospital, University of Copenhagen, Copenhagen, Denmark; 175 Unit of Medical Genetics, Department of Preventive and Predictive Medicine, Fondazione IRCCS Istituto Nazionale dei Tumori (INT), Milan, Italy; 176 Division of Cancer Prevention and Genetics, Istituto Europeo di Oncologia (IEO), Milan, Italy; 177 Department of Experimental Oncology, Istituto Europeo di Oncologia (IEO), Milan, Italy and Cogentech Cancer Genetic Test Laboratory, Milan, Italy; 178 University of Kansas Medical Center, Kansas City, KS, USA; 179 Department of Medical Oncology, Mayo Clinic, Rochester, Minnesota, USA; 180 College of Pharmacy and Health Sciences, Texas Southern University, Houston, Texas, USA; 181 Department of Gynecologic Oncology, The University of Texas MD Anderson Cancer Center, Houston, Texas, USA; 182 Department of Epidemiology, The University of Texas MD Anderson Cancer Center, Houston, Texas, USA; 183 Gynecology Service, Department of Surgery, Memorial Sloan-Kettering Cancer Center, New York, NY, USA; 184 Department of Obstetrics and Gynecology, Duke University Medical Center, Durham, North Carolina, USA; 185 Department of Statistical Science, Duke University, Durham, North Carolina, USA; 186 Department of Surgery, Duke University Medical Center, Durham, North Carolina, USA; 187 Cancer Prevention, Detection & Control Research Program, Duke Cancer Institute, Durham, North Carolina, USA; 188 Obstetrics and Gynecology Epidemiology Center, Brigham and Women's Hospital, Boston, Massachusetts, USA; 189 Channing Division of Network Medicine, Brigham and Women's Hospital and Harvard Medical School; 190 Department of Epidemiology, Harvard TH Chan School of Public Health, Boston, Massachusetts, USA; 191 Cancer Prevention and Control Program, Rutgers Cancer Institute of New Jersey, The State University of New Jersey, New Brunswick, NJ, USA; 192 Department of Epidemiology and Biostatistics, Memorial Sloan Kettering Cancer Center, New York, NY, USA; 193 Department of Gynecology and Obstetrics, Haukeland University Horpital, Bergen, Norway; 194 Centre for Cancer Biomarkers, Department of Clinical Sciences, University of Bergen, Bergen, Norway; 195 Radboud university medical center, Department of Gynaecology, Nijmegen, Netherlands; 196 Radboud university medical centre, Radboud Institute for Health Sciences, Nijmegen, Netherlands; 197 Netherlands Comprehensive Cancer Organisation, Utrecht, Netherlands; 198 Department of Obstetrcs & Gynecology, Oregon Health & Science University; 199 Knight Cancer Institute, Oregon Health & Science University, Portland, Oregon, USA; 200 Canada's Michael Smith Genome Sciences Centre, BC Cancer Agency, Vancouver, BC, Canada; 201 Department of Biomedical Physiology and Kinesiology, Simon Fraser University, Burnaby, BC Canada; 202 Department of Public Health Sciences, College of Medicine, Medical University of South Carolina, SC, USA; 203 Hollings Cancer Center, Medical University of South Carolina, SC, USA; 204 Division of Epidemiology and Biostatistics, Department of Internal Medicine, University of New Mexico, Albuquerque, New Mexico, USA; 205 Cancer Control Research, BC Cancer Agency, Vancouver, BC, Canada; 206 International Hereditary Cancer Center, Department of Genetics and Pathology, Pomeranian Medical University, Szczecin, Poland; 207 Department of Gynecological Surgery and Gynecological Oncology of Adults and Adolescents, Pomeranian Medical University, Szczecin, Poland; 208 Gyn Clinic, Rigshospitalet, University of Copenhagen, Denmark; 209 Department of Pathology, Rigshospitalet, University of Copenhagen, Denmark; 210 Department of Oncology, Rigshospitalet, University of Copenhagen, Denmark; 211 Department of Oncology, University of Cambridge, Strangeways Research laboratory, Cambridge, UK; 212 Cancer Genetics Laboratory, Research Division, Peter MacCallum Cancer Centre, St Andrews Place, East Melbourne; 213 Institute of Cancer Sciences, University of Glasgow, Wolfson Wohl Cancer Research Centre, Beatson Institute for Cancer Research, Glasgow, UK; 214 The Cancer Research UK Clinical Trials Unit, Beatson West of Scotland Cancer Centre, 1053 Great Western Road, Glasgow, G12 0YN; 215 Department of Gynaecological Oncology, Glasgow Royal Infirmary; 216 Department of Health Research and Policy - Epidemiology, Stanford University School of Medicine, Stanford CA, USA; 217 Epidemiology Center, College of Medicine, University of South Florida, Tampa, Florida, USA; 218 Public Health Ontario, Toronto, Canada; 219 Women's College Research Institute, University of Toronto, Toronto, Ontario, Canada; 220 Department of Cancer Epidemiology, Moffitt Cancer Center, Tampa, FL, USA; 221 Department of Biostatistics and Bioinformatics, Moffitt Cancer Center, Tampa, FL, USA; 222 Women's Cancer, Institute for Women's Health, UCL, London, United Kingdom; 223 Department of Preventive Medicine, Keck School of Medicine, University of Southern California Norris Comprehensive Cancer Center, Los Angeles, California, USA; 224 Department of Epidemiology and Biostatistics, Memorial Sloan-Kettering Cancer Center, New York, New York, USA; 225 Department of Pathology and Laboratory Diagnostics, The Maria Sklodowska-Curie Memorial Cancer Center and Institute of Oncology, Warsaw, Poland; 226 Department of Medicine, The University of Melbourne Health, Australia,; 227 The Royal Melbourne Hospital, Victoria 3050, Australia; 228 Cancer Epidemiology Centre, Cancer Council Victoria, Victoria, Australia

**Keywords:** Cancer: breast, Cancer: prostate, Genetics, Cancer: ovary, cancer predisposition

## Abstract

**Background:**

The rarity of mutations in *PALB2*, *CHEK2* and *ATM* make it difficult to estimate precisely associated cancer risks. Population-based family studies have provided evidence that at least some of these mutations are associated with breast cancer risk as high as those associated with rare *BRCA2* mutations. We aimed to estimate the relative risks associated with specific rare variants in *PALB2*, *CHEK2* and *ATM* via a multicentre case-control study.

**Methods:**

We genotyped 10 rare mutations using the custom iCOGS array: *PALB2* c.1592delT, c.2816T>G and c.3113G>A, *CHEK2* c.349A>G, c.538C>T, c.715G>A, c.1036C>T, c.1312G>T, and c.1343T>G and *ATM* c.7271T>G. We assessed associations with breast cancer risk (42 671 cases and 42 164 controls), as well as prostate (22 301 cases and 22 320 controls) and ovarian (14 542 cases and 23 491 controls) cancer risk, for each variant.

**Results:**

For European women, strong evidence of association with breast cancer risk was observed for *PALB2* c.1592delT OR 3.44 (95% CI 1.39 to 8.52, p=7.1×10^−5^), *PALB2* c.3113G>A OR 4.21 (95% CI 1.84 to 9.60, p=6.9×10^−8^) and *ATM* c.7271T>G OR 11.0 (95% CI 1.42 to 85.7, p=0.0012). We also found evidence of association with breast cancer risk for three variants in *CHEK2*, c.349A>G OR 2.26 (95% CI 1.29 to 3.95), c.1036C>T OR 5.06 (95% CI 1.09 to 23.5) and c.538C>T OR 1.33 (95% CI 1.05 to 1.67) (p≤0.017). Evidence for prostate cancer risk was observed for *CHEK2* c.1343T>G OR 3.03 (95% CI 1.53 to 6.03, p=0.0006) for African men and *CHEK2* c.1312G>T OR 2.21 (95% CI 1.06 to 4.63, p=0.030) for European men. No evidence of association with ovarian cancer was found for any of these variants.

**Conclusions:**

This report adds to accumulating evidence that at least some variants in these genes are associated with an increased risk of breast cancer that is clinically important.

## Introduction

The rapid introduction of massive parallel sequencing (MPS) into clinical genetics services is enabling the screening of multiple breast cancer susceptibility genes in one assay at reduced cost for women who are at increased risk of breast (and other) cancer. These gene panels now typically include the so-called ‘moderate-risk’ breast cancer susceptibility genes, including *PALB2*, *CHEK2* and *ATM.*^1–3^ However, mutations in these genes are individually extremely rare and limited data are available with which to accurately estimate the risk of cancer associated with them.

Estimation of the age-specific cumulative risk (penetrance) of breast cancer associated with specific mutations in these three genes has been limited to those that have been observed more frequently, such as *PALB2* c.1592delT (a Finnish founder mutation), *PALB2* c.3113G>A and *ATM* c.7271T>G. These mutations have been estimated to be associated with a 40% (95% CI 17% to 77%), 91% (95% CI 44% to 100%) and 52% (95% CI 28% to 80%) cumulative risk of breast cancer to the age of 70 years, respectively.^4–7^ These findings, based on segregation analyses in families of population-based case series, indicate that at least some mutations in these ‘moderate-risk’ genes are associated with a breast cancer risk comparable to that of the average pathogenic mutation in *BRCA2*: 45% (95% CI 31% to 56%).^8^ However, such estimates are imprecise and, moreover, may be confounded by modifying genetic variants or other familial risk factors.

Case-control studies provide an alternative approach to estimating cancer risks associated with specific variants. This design can estimate the relative risk directly, without making assumptions about the modifying effects of other risk factors. However, because these variants are rare, such studies need to be extremely large to provide precise estimates.

The clearest evidence for association, and the most precise breast cancer risk estimates, for rare variants in *PALB2, CHEK2* and *ATM* relate to protein truncating and splice-junction variants.^9^ ^10^ However, studies based on mutation screening in case-control studies, combined with stratification of variants by their evolutionary likelihood suggest that at least some evolutionarily unlikely missense substitutions are associated with a similar risk to those conferred by truncating mutations.^11–13^ For example, Tavtigian *et al*^12^ estimated an OR of 2.85 (95% CI 0.83 to 4.86) for evolutionarily unlikely missense substitutions in the 3′ third of *ATM*, which is comparable to that for truncating variants. Specifically, *ATM* c.7271C>G has been associated with a more substantial breast cancer risk in several studies.^7^ ^13^ Le Calvez-Kelm *et al*,^11^ estimated that the ORs associated with rare mutations in *CHEK2* from similarly designed studies were 6.18 (95% CI 1.76 to 21.8) for rare protein-truncating and splice-junction variants and 8.75 (95% CI 1.06 to 72.2) for evolutionarily unlikely missense substitutions.^11^

It is plausible that monoallelic mutations in *PALB2*, *CHEK2* and *ATM* could be associated with increased risk of cancers other than breast cancer, as has been observed for *BRCA1* and *BRCA2* and both ovarian and prostate cancers.^14–17^ However, with the exception of pancreatic cancer in *PALB2* carriers, there is little evidence to support or refute the existence of such associations, although a few individually striking pedigrees have been observed.^4^ ^8^ ^18–20^

In this study we selected rare genetic variants on the basis that they had been observed in breast cancer candidate gene case-control screening projects involving *PALB2*, *CHEK2* or *ATM*. These included three rare variants in *PALB2*: the protein truncating variants c.1592delT (p.Leu531Cysfs)^4^ and c.3113 G>A (p.Trp1038*)^6^ and the missense variant c.2816T>G, (p.Leu939Trp), six rare missense variants in CHEK2: c.349A>G (p.Arg117Gly) and c.1036C>T (p.Arg346Cys) predicted to be deleterious on the basis of evolutionary conservation,^11^ c.538C>T (p.Arg180Cys), c.715G>A (p.Glu239Lys), c.1312G>T (p.Asp438Tyr) and c.1343T>G (p.Ile448Ser) and *ATM* c.7271T>G (p.Val2424Gly).^7^ We assessed the association of these variants with breast, ovarian and prostate risk by case-control analyses in three large consortia participating in the Collaborative Oncological Gene-environment Study.^21^ ^22^

## Methods

### Participants

Participants were drawn from studies participating in three consortia as follows:

The *Breast Cancer Association Consortium (BCAC),* involving a total of 48 studies: 37 of women from populations with predominantly European ancestry (42 671 cases and 42 164 controls), 9 of Asian women (5795 cases and 6624 controls) and 2 of African-American women (1046 cases and 932 controls). All cases had invasive breast cancer. The majority of studies were population-based or hospital-based case-control studies, but some studies of European women oversampled cases with a family history or with bilateral disease (see online supplementary table S1). Overall, 79% of BCAC cases with known Estrogen Recptor (ER) status (23% missing) are ER-positive. The proportion of cases selected by family history that are ER-positive is 78% (38% missing).

10.1136/jmedgenet-2016-103839.supp1 Supplementary data

The *Prostate Cancer Association Group to Investigate Cancer Associated Alterations in the Genome (PRACTICAL)* involving a total of 26 studies: 25 included men with European ancestry (22 301 cases and 22 320 controls) and 3 included African-American men (623 cases and 569 controls). The majority of studies were population-based or hospital-based case-control studies (see online supplementary table S2).

The *Ovarian Cancer Association Consortium (OCAC)*, involving a total of 46 studies. Some studies were case-only and their data were combined with case-control studies from the same geographical region (leaving 36 study groupings). Of these groupings, 33 included women from populations with predominantly European ancestry (16 287 cases (14 542 with invasive disease) and 23 491 controls), 25 included Asian women (813 cases (720 with invasive disease) and 1574 controls), 17 included African-American women (186 cases (150 with invasive disease) and 200 controls) and 29 included women of other ethnic origin (893 cases (709 with invasive disease) and 864 controls). The majority of studies were population-based or hospital-based case-control studies (see online supplementary table S3).

Details regarding sample quality control have been published previously.^22^ ^23^ All study participants gave informed consent and all studies were approved by the corresponding local ethics committees (see online supplementary tables S1–S3).

### Variant selection

We selected for genotyping 13 rare mutations that had been observed in population-based case-control mutation screening studies. These variants were *PALB2* (c.1592delT, p.Leu531Cysfs;^4^ ^5^ ^10^ c.2323C>T p.Gln775*;^20^ c.2816T>G, p.Leu939Trp;^2^ ^20^ c.3113G>A, p.Trp1038*;^2^ ^6^ ^20^ c.3116delA, p.Asn1039IIefs;^2^ ^6^ ^20^ c.3549C>G, p.Tyr1183*^2^), *CHEK2* (c.349A>G, p.ArgR117Gly; c.538C>T, p.Arg180Cys; c.715G>A p.Glu239Lys; c.1036C>T, p.Arg346Cys; c.1312G>T, p.Asp438Tyr; c.1343T>G, p.Ile448Ser)^11^ and *ATM* (c.7271T>G, p.Val2424Gly)^7^ ^13^ ^24^ see table 1. A DNA sample carrying each of these variants was included in a plate of control DNAs that was distributed to each genotyping centre to assist with quality control and genotype calling.

**Table 1:** Rare genetic variants included in the iCOGS array.

Gene	Variant*	Amino acid*	dbSNP rs	Breast cancer risk estimates		Align-GVGD	Reference(s)	Designed‡	Genotyped
				OR (95% CI)	Penetrance† (95% CI)				
*PALB2*	c.1592delT	p.Leu531Cysfs	rs180177102	3.94 (1.5-12.1)§	40% (17–77)	na	4^,^ 5^,^ 10	Yes	Yes
	c.2323C>T	p.Gln775*	rs180177111			na	25^,^ 26	No	No
	c.2816T>G	p.Leu939Trp	rs45478192			C55	20	Yes	Yes
	c.3113G>A	p.Trp1038*	rs180177132		95% (44–100)	na	2^,^ 6^,^ 20	Yes	Yes
	c.3116delA	p.Asn1039Ilefs	rs180177133			na	2	No	No
	c.3549C>G	p.Tyr1183*	rs118203998			na	2	No	No
CHEK2	c.349A>G	p.Arg117Gly	rs28909982	8.75 (1.06–72.2)¶		C65	11	Yes	Yes
	c.538C>T	p.Arg180Cys	rs77130927	2.47 (0.45–13.49)**		C25	11	Yes	Yes
	c.715G>A	p.Glu239Lys	rs121908702	1.82 (0.62–5.34)††		C15	11	Yes	Yes
	c.1036C>T	p.Arg346Cys	na	8.75 (1.06–72.2)¶		C65	11	Yes	Yes
	c.1312G>T	p.Asp438Tyr	na	2.47 (0.45–13.49)**		C25	11	Yes	Yes
	c.1343T>G	p.Ile448Ser	rs17886163	1.82 (0.62–5.34)††		C15	11	Yes	Yes
ATM	c.7271T>G	p.Val2424Gly	rs28904921		52% (28–80)	C65	7^,^ 13^,^ 23^,^ 27	Yes	Yes

*Human Genome Variation Society (HGVS); reference sequences PALB2, NM_024675.3, NP_078951.2; CHEK2, NM_007194.3, NP_009125.1; ATM, NM_000051.3, NP_000042.3.

†Age-specific cumulative risk of breast cancer to age 70 years.5–7

‡Able to be designed for measurement on the custom Illumina iSelect genotyping array.^21^ ^22^

**§**Breast cancer cases unselected for family history of breast cancer.^4^

¶OR estimated in a combined group of C65 CHEK2 variants.^11^

**OR estimated in a combined group of C25 CHEK2 variants.^11^

††OR estimated in a combined group of C15 CHEK2 variants.^11^

na, not available.

### Genotyping

Three *PALB2* variants c.2323C>T (p.Gln775*), c.3116delA (p.Asn1039IIefs) and c.3549C>G (p.Tyr1183*) were unable to be designed for measurement on the custom Illumina iSelect genotyping array and were not considered further (table 1). Genotyping was conducted using a custom Illumina Infinium array (iCOGS) in four centres, as part of a multiconsortia collaboration as described previously.^22^ Genotypes were called using Illumina's proprietary GenCall algorithm and then, for the data generated from the rare variant probes, manually confirmed with reference to the positive control sample. Two per cent of samples were provided in duplicate by all studies and 270 HapMap2 samples were genotyped in all four genotyping centres. Subjects with an overall call rate <95% were excluded. Plates with call rates <90% were excluded on a variant-by-variant basis. Cluster plots generated for all of the 10 rare variants were manually checked to confirm automated calls (see online supplementary figure S1).

### Statistical methods

The association of each variant with breast, prostate and ovarian cancer risk was assessed using unconditional logistic regression to estimate ORs for carriers versus non-carriers, adjusting for study (categorical). p Values were determined by the likelihood ratio test comparing models with and without carrier status as a covariate. We also applied conditional logistic regression, defining risk sets by study, and found that this made no difference to the OR estimates, CIs or p values to two significant figures; since model convergence was a problem for this latter regression analysis, all subsequent analyses were based on unconditional logistic regression. For the main analyses of breast cancer risk in European women, we also included as covariates the first six principal components, together with a seventh component specific to one study (Leuven Multidisciplinary Breast Centre (LMBC)) for which there was substantial inflation not accounted for by the components derived from the analysis of all studies. Addition of further principal components did not reduce inflation further. Data from all breast cancer studies were included to assess statistical significance. Data from cases selected for inclusion based on personal or family history of breast cancer were excluded in order to obtain unbiased OR estimates for the general population of white European women (leaving 37 039 cases and 38 260 controls from 32 studies). Multiple testing was adjusted for using the Benjamini-Hochberg procedure to control the false discovery rate, with a significance threshold of 0.05.^25^ Reported p values are unadjusted unless otherwise stated. Reported CIs are all nominal. We included two race-specific principal components in each of the main breast cancer analyses of Asian and African-American women. Similar analyses were conducted using the data from PRACTICAL and OCAC, consistent with those used previously.^23^ ^26^ All analyses were carried out using Stata: Release V.10 (StataCorp, 2008).

## Results

### PALB2

In BCAC, *PALB2* c.1592delT (Leu531Cysfs) was only observed in 35 cases and 6 controls, all from four studies from Sweden and Finland (Helsinki Breast Cancer Study (HEBCS), Kuopio Breast Cancer Project (KBCP), Oulu Breast Cancer Study (OBCS) and Karolinska Mammography Project for Risk Prediction Breast Cancer (pKARMA); see online supplementary table S1), giving strong evidence of association with breast cancer risk (p=7.1×10^−5^); the OR estimate was 4.52 (95% CI 1.90 to 10.8) based on all studies and 3.44 (95% CI 1.39 to 8.52) based on unselected cases and controls (table 2). We also found evidence of heterogeneity by ER status (p=0.0023), the association being stronger for ER-negative disease (OR 6.49 (95% CI 2.17 to 19.4) versus 2.24 (95% CI 1.05 to 7.24) for ER-positive disease).

**Table 2:** Summary results from Breast Cancer Association Consortium studies of white Europeans **(**42 671 invasive breast cancer cases and 42 164 controls)

Variant	Frequency* Controls	Frequency* Cases	OR (95% CI)	LRT p Value	OR† (95% CI)	LRT p Value†
*PALB2*§						
**c.1592delT (p.Leu531Cysfs)**	0.00014	0.00082	4.52 (1.90 to 10.8)	7.1×10^−5^	3.44 (1.39 to 8.52)	0.003
c.2816T>G (p.Leu939Trp)	0.00342	0.00352	1.05 (0.83 to 1.32)	0.70	1.03 (0.80 to 1.32)	0.82
c.3113G>A (p.Trp1038*)	0.00019	0.00101	5.93 (2.77 to 12.7)	6.9×10^−8^	4.21 (1.84 to 9.60)	1.2×10^−4^
***CHEK2***						
**c.349A>G (p.Arg117Gly)**	0.00043	0.00103	2.26 (1.29 to 3.95)	0.003	2.03 (1.10 to 3.73)	0.020
**c.538C>T (p.Arg180Cys)**	0.00337	0.00370	1.33 (1.05 to 1.67)	0.016	1.34 (1.06 to 1.70)	0.015
c.715G>A (p.Glu239Lys)	0.00021	0.00035	1.70 (0.73 to 3.93)	0.210	1.47 (0.60 to 3.64)	0.40
c.1036C>T (p.Arg346Cys)	0.00005	0.00021	5.06 (1.09 to 23.5)	0.017	3.39 (0.68 to 16.9)	0.11
c.1312G>T (p.Asp438Tyr)	0.00078	0.00082	1.03 (0.62 to 1.71)	0.910	0.87 (0.49 to 1.52)	0.62
c.1343T>G (p.Ile448Ser)‡	0.00002	0	–	–	–	–
***ATM***						
**c.7271T>G (p.Val2424Gly)**	0.00002	0.00028	11.6 (1.50 to 89.9)	0.0012	11.0 (1.42 to 85.7)	0.0019

*Proportion of subjects carrying the variant.

†Excluding women from five studies that selected all cases based on family history or bilateral disease and the subset of selected cases from other studies (based on 34 488 unselected cases and 34 059 controls).

‡*CHEK2* c.1343T>G (p.Ile448Ser) was only observed in one control and no cases of white European origin.

§*PALB2* c.3113G>A (p.Trp1038*) only observed in the UK, Australia, the USA and Canada. *PALB2* c.1592delT (p.Leu531Cysfs) only observed in Finland and Sweden.

LRT, likelihood ratio test; OR, OR for carriers of the variant versus common-allele homozygotes, adjusted for study and seven principal components.

*PALB2* c.3113G>A (p.Trp1038*) was identified in 44 cases and 8 controls from nine BCAC studies. Only one carrier of the variant was of non-European origin. Strong evidence of association with breast cancer risk was observed (p=6.9×10^−8^), with an estimated OR of 5.93 (95% CI 2.77 to 12.7) based on all studies and 4.21 (95% CI 1.85 to 9.61) based on unselected cases and controls. There was no evidence of a differential association by ER status (p=0.15).

Based on unselected cases, the estimated OR associated with carrying either of these *PALB2* variants (c.1592delT or c.3113G>A) was 3.85 (95% CI 2.09 to 7.09).

*PALB2* c.2816T>G (p.Leu939Trp) was identified in 150 cases and 145 controls and there was no evidence of association with risk of breast cancer. There was no evidence of association with risk of prostate or ovarian cancer for any of the three *PALB2* variants (see tables 3 and 4).

**Table 3:** Summary results from the Prostate Cancer Association Group to Investigate Cancer Associated Alterations in the Genome studies for white European men* (22 301 prostate cancer cases and 22 320 controls)

Variant	Frequency† Controls	Frequency† Cases	OR (95% CI)	LRT p Value
*PALB2*				
c.1592delT (p.Leu531Cysfs)	0.00018	0.00031	2.06 (0.59 to 7.11)	0.24
c.2816T>G (p.Leu939Trp)	0.00354	0.00381	0.95 (0.69 to 1.29)	0.73
c.3113G>A (p.Trp1038*)	0.00045	0.00027	0.49 (0.18 to 1.36)	0.16
*CHEK2*‡				
c.349A>G (p.Arg117Gly)	0.00063	0.00081	1.46 (0.71 to 3.02)	0.30
c.538C>T (p.Arg180Cys)	0.00341	0.00296	1.02 (0.73 to 1.44)	0.90
c.715G>A (p.Glu239Lys)	0.00018	0.00027	1.47 (0.41 to 5.35)	0.55
c.1036C>T (p.Arg346Cys)	0.00018	0.00022	1.07 (0.28 to 4.07)	0.93
c.1312G>T (p.Asp438Tyr)	0.00049	0.00103	2.21 (1.06 to 4.63)	0.03
c.1343T>G (p.Ile448Ser)	0	0.00009	–	–
c.1343T>G (Africans§)	0.019	0.057	3.03 (1.53 to 6.03)	0.001
ATM				
c.7271T>G (p.Val2424Gly)	0.00004	0.00027	4.37 (0.52 to 36.4)	0.17

*For white European men, unless otherwise indicated.

†Proportion of subjects carrying the variant.

‡*CHEK2* c.1343T>G (p.Ile448Ser) was the only *CHEK2* variant observed in African men and was identified in two cases and no controls of white European origin.

§Based on data from 623 and 569 African-American cases and controls, respectively.

LRT, likelihood ratio test; OR, OR for carriers of the variant versus common-allele homozygotes, adjusted for study and seven principal components.

**Table 4:** Summary results from the Ovarian Cancer Association Consortium studies for white European women (14 542 invasive ovarian cancer cases and 23 491 controls)

Variant	Frequency* Controls	Frequency* Cases	OR (95% CI)	LRT p Value
*PALB2*				
c.1592delT (p.Leu531Cysfs)	0.00004	0.00012	2.50 (0.21 to 29.1)	0.45
c.2816T>G (p.Leu939Trp)	0.00413	0.00399	0.96 (0.69 to 1.34)	0.81
c.3113G>A (p.Trp1038*)	0.00034	0.00031	1.34 (0.36 to 4.97)	0.66
*CHEK2*				
c.349A>G (p.Arg117Gly)	0.00038	0.00031	1.07 (0.32 to 3.60)	0.92
c.538C>T (p.Arg180Cys)	0.00128	0.00160	1.49 (0.83 to 2.67)	0.18
c.715G>A (p.Glu239Lys)	0.00021	0.00037	1.47 (0.42 to 5.22)	0.54
c.1036C>T (p.Arg346Cys)‡	0	0	–	–
c.1312G>T (p.Asp438Tyr)	0.00081	0.00074	0.92 (0.42 to 1.99)	0.83
c.1343T>G (p.Ile448Ser)	0.00009	0	–	–
*ATM*				
c.7271T>G (p.Val2424Gly)	0	0.00012	–	–

*Proportion of subjects carrying the variant.

‡c.1036C>T (p.Arg346Cys) was not observed in any sample.

LRT, likelihood ratio test; OR, OR for carriers of the variant versus common-allele homozygotes, adjusted for study and seven principal components.

### CHEK2

*CHEK2* c.349A>G (p.Arg117Gly) was identified in 44 cases and 18 controls in studies participating in BCAC; all of these women were of European origin. We found evidence of association with breast cancer (p=0.003), with little change in the OR after excluding selected cases (OR 2.03 (95% CI 1.10 to 3.73)).

*CHEK2* c.538C>T (p.Arg180Cys) was identified in 158 breast cancer cases and 142 controls in studies of white Europeans. Evidence of association with breast cancer risk (p=0.016) was observed, with an unbiased OR estimate of 1.34 (95% CI 1.06 to 1.70). A consistent OR estimate was observed for Asian women, based on 45 case and 45 control carriers (OR 1.16 (95% CI 0.75 to 1.76)).

*CHEK2* c.715G>A (p.Glu239Lys) mutations were identified in 15 cases and 9 controls, all European women participating in BCAC and no evidence of association with risk of breast cancer was observed (p=0.21).

*CHEK2* c.1036C>T (p.Arg346Cys) was identified in nine cases from seven studies and two controls from two different studies in BCAC (neither control carrier was from a study that had case carriers), all of European origin. We found evidence of association with breast cancer risk (p=0.017) with reduced OR estimate of 3.39 (95% CI 0.68 to 16.9) after excluding selected cases.

None of the above four *CHEK2* variants (*CHEK2* c.349A>G (p.Arg117Gly); c.538C>T (p.Arg180Cys); c.715G>A (p.Glu239Lys) and c.1036C>T (p.Arg346Cys)) were found to be associated with an increased risk of prostate or ovarian cancer (tables 3 and 4). *CHEK2* variant c.1312G>T (p.Asp438Tyr) was not associated with risk of breast cancer for European women (p=0.91). Variant c.1343T>G (p.Ile448Ser) was not observed in any breast cancer cases of European or Asian origin. It was detected in 48 cases and 29 controls of African origin, giving weak evidence of association (OR 1.52 (95% CI 0.95 to 2.43, p=0.083)). *CHEK2* c.1312G>T (p.Asp438Tyr) was identified in 23 cases and 11 controls from PRACTICAL, all European, providing evidence of association with prostate cancer risk (OR 2.21 (95% CI 1.06 to 4.63, p=0.030)). *CHEK2* c.1343T>G (p.Ile448Ser) was observed in 35 cases and 11 controls, all African, participating in PRACTICAL and was also associated with an increased risk of prostate cancer (OR 3.03 (95% CI 1.53 to 6.03, p=0.00059)). There was no evidence that these *CHEK2* variants were associated with risk of ovarian cancer (table 4).

### ATM

ATM c.7271T>G (p.Val2424Gly) was identified in 12 cases and 1 control in studies participating in BCAC, all of European origin, giving evidence of association with breast cancer risk (p=0.0012). The OR estimate based on unselected studies was 11.0 (95% CI 1.42 to 85.7). There was no evidence of association of this variant with prostate or ovarian cancer risk (see tables 3 and 4).

## Discussion

The present report adds to an accumulating body of evidence that at least some *rare variants* in so-called ‘moderate-risk’ genes are associated with an increased risk of breast cancer that is of clinical relevance.

These findings are presented at a time when detailed information about variants in these genes is becoming more readily available via the translation of diagnostic genetic testing from Sanger sequencing-based testing platforms to MPS platforms that test panels of genes in single assays.^27–29^ The vast majority of information about *PALB2*, *CHEK2* and *ATM*, variants generated from these new testing platforms is not being used in clinical genetics services due to lack of reliable estimates of the cancer risk associated with individual variants, or groups of variants, in each gene. Previous analyses have been largely based on selected families, relying on data on the segregation of the variant. The present study is by far the largest to take a case-control approach. Consistent with previous reports,^5–7^ ^9^ ^11–13^ *PALB2* c.3113G>A (p.Trp1038*), *PALB2* c.1592delT (p.Leu531Cysfs) and *ATM* c.7271T>G (p.Val2424Gly) were found to be associated with substantially increased risk of breast cancer all with associated relative risk estimates of 3.44 or greater.

The estimates for the two loss-of-function *PALB2* variants (c.1592delT and c.3113G<A) were consistent with each other and with estimates based on segregation analysis.^5^ ^6^ ^9^ We found no evidence of association with breast cancer for *PALB2* c.2816T>G (p.Leu939Trp), with an upper 95% confidence limit excluding an OR >1.5 which is notable given the Align-Grantham Variation Granthan Deviation (Align-GVGD) score and the observed impact on protein function.^30^

The estimate for *ATM* c.7271T>G (p.Val2424Gly) was also consistent with that found by segregation analysis.^7^ ^13^ The substantial increased risk of breast cancer associated with *ATM* c.7271T>G (p.Val2424Gly) could be due to the reduction in kinase activity (with near-normal protein levels) observed for ATM p.Val2424Gly,^31^ thus this variant is likely to be acting as a dominant negative mutation.^32^

In contrast, we found no evidence of an association with risk of prostate or ovarian cancer with any of these three variants: however, the confidence limits were wide; based on the upper 95% confidence limit we could exclude an OR of >1.4 for prostate cancer for the loss-of-function *PALB2* c.3113G>A and 1.9 for c.1592delT and c.3113G>A combined.

We analysed six rare missense variants in *CHEK2*. Two of these (*CHEK2* c.349A>G (p.Arg117Gly; rs28909982) and c.1036C>T (p.Arg346Cys)) had evidence of a significant impact on the protein based on in silico prediction. We proposed these variants for inclusion in the iCOGS design as they had been identified in 3/1242 cases and 1/1089 controls and 3/1242 cases and 0/1089 controls, respectively, in a population-based case-control mutation screening study of CHEK2.^11^ In that study, Le Calvez-Kelm *et al*, estimated an OR of 8.75 (95% CI 1.06 to 72.2) for variants with an Align-GVGD score C65 (based on nine cases and one control). The current analysis provides confirmatory evidence of this association in a much larger sample (OR 2.18 (95% CI 1.23 to 3.85)) including 40 unselected case and 18 control carriers. The evidence that *CHEK2* is a breast cancer susceptibility gene is largely based on studies of protein truncating variants, in particular *CHEK2* 1100delC.^33^ Reports of the association of the missense variant I157T, (C15) and breast cancer risk have been conflicting but a large meta-analysis involving 15 985 breast cancer cases and 18 609 controls estimated a modest OR of 1.58 (95% CI 1.42 to 1.75).^34^ We also found evidence (p=0.015) of an association for c.538C>T (Align-GVGD C25); OR 1.34 (95% CI 1.06 to 1.70), a risk comparable to I157T.

The p values reported above have not been adjusted for multiple testing. This was not considered appropriate for the associations with breast cancer risk of *PALB2* c.1592delT, c.3113G>A and *ATM* c.7271T>G because these associations had previously been reported; our aim was to more precisely estimate the associated relative risks. All three associations with breast cancer risk reported for *CHEK2* variants remained statistically significant after adjusting for the other tests conducted in relation to breast cancer risk, but not after correcting for all tests for all cancers. Nevertheless, the findings for *CHEK2* c.349A>G and c.1036C>T confirmed those reported previously, although collectively. The association observed with *CHEK2* c.538C>T requires independent replication.

Do this approach and new data have an impact on clinical recommendations for women and families carrying these rare genetic variants? Although age-specific cumulate risks for cancer are more informative for genetic counselling and clinical management of carriers, our study provides information that is relevant to clinical recommendations. As discussed in Easton *et al*,^35^ a relative risk of 4 will place a woman in a ‘high-risk’ category (in the absence of any other risk factor) and a relative risk between 2 and 4 will place a woman in this category if other risk factors are present. Thus, several of the variants included in this report (*PALB2* c.1592delT; c.3113G>A *ATM* c.7271T>G) would place the carrier in a high-risk group, especially if other risk factors, such as a family history, are present. The high level of breast cancer risk associated with *PALB2* c.1592delT and c.3113G>A reported here is consistent with the penetrance estimate reported for a group of loss-of-function mutations in *PALB2*^9^ and has an advantage in terms of clinical utility that the estimates in this study have been made at a mutation-specific level. Therefore, this work provides important information for risk reduction recommendations (such as prophylactic mastectomy and potentially salpingo-oophorectomy) for carriers of these variants. However, further prospective research is required to characterise these risks and to understand the potential of other risk-reducing strategies such as salpingo-oophorectomy and chemoprevention.

The consistency of the relative risk estimates with those derived through family based studies supports the hypothesis that these variants combine multiplicatively with other genetic loci and familial risk factors; this information is critical for deriving comprehensive risk models. Even with very large sample sizes such as those studied here, however, it is still only possible to derive individual risk estimates for a limited set of variants, and even for these variants the estimates are still imprecise. This internationally collaborative approach also has limited capacity to improve risk estimates for rare variants that are only observed in specific populations. Inevitably, therefore, risk models will depend on combining data across multiple variants, using improved in silico predictions and potentially biochemical/functional evidence to synthesise these estimates efficiently. It will also be necessary develop counselling and patient management strategies that can accommodate a multifactorial approach to variant classification.

10.1136/jmedgenet-2016-103839.supp2 Supplementary Financial Support
