# Supplementary material for: PALB2, CHEK2 and ATM rare variants and cancer risk: data from COGS
Source: J Med Genet. 2016 Sep 5;53(12):800–11. doi: 10.1136/jmedgenet-2016-103839 (PMC5200636; doi:10.1136/jmedgenet-2016-103839)

Supplementary Table 1. Description of the BCAC studies contributing to COGS.

Study Acronym	Study Name	Country	Recruitment	
			**Cases**	**Controls**
ABCFS†	Australian Breast Cancer Family Study [1]	Australia	Cancer registries in Victoria and New South Wales (1992-1999): all cases from Melbourne and Sydney diagnosed before age 40 plus a random sample of those diagnosed at ages 40-59.	Identified between 1992 and 1999 from the electoral rolls in Melbourne and Sydney (enrolling to vote is compulsory); frequency matched to cases by age in-5 year categories.
ABCS	Amsterdam Breast Cancer Study [2]	Netherlands	Breast cancer patients diagnosed before age 50 in 2003-2009 at the NKI-AVL; and (ABCS-F) non-*BRCA1/2* breast cancer cases from the family cancer clinic of the NKI-AVL tested in the period 1995-2009, of all ages and diagnosed with breast cancer in 1965-2008.	Population-based cohort of women recruited through the Sanquin blood bank, all ages.
BBCC	Bavarian Breast Cancer Cases and Controls [3]	Germany	Consecutive, unselected cases with invasive breast cancer recruited at the University Breast Centre, Franconia in Northern Bavaria from 2002-2010.	Healthy women aged 55 or older with no diagnosis of cancer. Invited by a newspaper advertisement in Northern Bavaria between 2002-2010.
BBCS	British Breast Cancer Study [4]	U.K.	(i) English & Scottish Cancer Registries: all breast cancer cases who developed a first primary before age 65 in 1971 or later and who subsequently developed a second primary cancer;<br>(ii) Unilateral breast cancer cases diagnosed before age 70 in 1971 or later.	A friend, sister-in-law, daughter-in-law or other non-blood relative of cases, recruited from 2001-2008.
BIGGS	Breast Cancer in Galway Genetic Study [5]	Ireland	Unselected cases recruited from University College Hospital Galway and surrounding hospitals in the West of Ireland since 2001	Women > 60 years with no personal history of any cancer and no family history of breast or ovarian cancer identified from retirement groups in the West of Ireland between 2001-2008.
BSUCH	Breast Cancer Study of the	Germany	All cases diagnosed with breast cancer in	Female blood donors recruited in 2007- 2009

	University Clinic Heidelberg [6]		2007-2009 at the University Women`s Clinic Heidelberg.	at the Institute of Transfusion Medicine & Immunology, Mannheim.
CECILE	CECILE Breast Cancer Study [7]	France	All cases diagnosed with breast cancer in 2005-2007 among women <75 years of age residing in the *départements* of Ille-et-Vilaine and Côte d'Or . Cases were recruited from the main cancer treatment center (Centre Eugène-Marquis in Rennes and Centre Georges-François-Leclerc in Dijon) and from other private or public hospitals in each area.	General population control women residing in the same areas as the cases (Ille-et-Vilaine and Côte d'Or). Controls were frequency-matched to the cases by 5-year age groups. They were recruited in 2005-2007 using a random digit dialing procedure and quotas by socioeconomic status to reflect the distribution by SES of the population in each area.
CGPS	Copenhagen General Population Study [8]	Denmark	Consecutive, incident cases from one hospital with centralized care for a population of 400,000 women in Copenhagen (2001-present).	Women with no history of breast cancer residing in the same region as cases identified from the Copenhagen General Population Study (2003-2007).
CNIO-BCS	Spanish National Cancer Centre Breast Cancer Study [9]	Spain	(i) consecutive breast cancer patients from three public hospitals, two in Madrid and one in Oviedo;<br>(ii) cases with at least one affected first degree relative recruited through the CNIO family cancer clinic in Madrid (2000-2005).	Women attending the Menopause Research Centre, Madrid and female members of the College of Lawyers attending a free, targeted medical check-up in Madrid between 2000 and 2005, all free of breast cancer.
CTS*	California Teachers Study [10]	U.S.A.	Nested case-control study conducted within a cohort of California teachers (113,590) who were under age 80 years at baseline, had no prior history of invasive or *in situ* breast cancer. Cases are women newly diagnosed with a histologically confirmed invasive primary adenocarcinoma of the breast at age 80 years or younger from 1998 to 2008.	Controls are a probability sample of at-risk cohort members, frequency matched to cases on age at baseline (5-year age groups), self-reported race/ethnicity (white, African American, Latina,Asian, other), and broad geographic region within California Controls were selected without replacement, using an assigned reference date.
ESTHER	ESTHER Breast Cancer Study [11]	Germany	Breast cancer cases in all hospitals in the state of Saarland, from 2001-2003 (ESTHER) and 1996-1998 (VERDI).	Random sample of women undergoing a routine health check-up in Saarland, in 2000-2002; frequency matched to cases by age in-5 year categories.
GENICA*	Gene Environment Interaction & Breast Cancer in Germany	Germany	Incident breast cancer cases enrolled at hospitals<br>in the Greater Bonn area between 2000-2004.	Random address sample selected in 2001-2004 from 31 population registries in the greater Bonn area; frequency matched to cases on

	[12,13]			year of birth in 5-year categories.
HEBCS	Helsinki Breast Cancer Study [14]	Finland	(1) Consecutive cases (883) from the Department of Oncology, Helsinki University Central Hospital 1997-8 and 2000, (2) Consecutive cases (986) from the Department of Surgery, Helsinki University Central Hospital 2001 – 2004, (3) Familial breast cancer patients (536) from the Helsinki University Central Hospital, Departments of Oncology and Clinical Genetics (1995-).	Healthy females from the same geographical region in Southern Finland in 2003.
HMBCS	Hannover-Minsk Breast Cancer Study [15]	Belarus	Cases from the Byelorussian Institute for Oncology and Medical Radiology Aleksandrov N.N. in Minsk or at one of 5 regional oncology centers in Gomel, Mogilev, Grodno, Brest or Vitebsk (2002-2008).	Women attending general medical examination at gynecology clinics in Gomel, Mogilev, Grodno, Brest or Vitebsk; women attending the Institute for Inherited Diseases in Minsk; female blood donors in Minsk; healthy relatives of cases (2002-2008).
KARBAC	Karolinska Breast Cancer Study [16]	Sweden	1. Familial cases from Department of Clinical Genetics, Karolinska University Hospital, Stockholm.<br>2. Consecutive cases from Department of Oncology, Huddinge & Söder Hospital, Stockholm 1998-2000.	Blood donors of mixed gender from same geographical region. Excess material was received from all blood donors over a 3 month period in 2004 (approximately 3000) and DNA was extracted from a random sample of 1500.
KBCP	Kuopio Breast Cancer Project [17]	Finland	Women seen at Kuopio University Hospital between 1990-1995 because of a breast lump, mammographic abnormality, or other breast symptom and who were found to have breast cancer.	Selected from the National Population Register between 1990-1995; age and long-term area-of-residence matched to cases.
kConFab/ AOCS	Kathleen Cuningham Foundation Consortium for Research into Familial Breast Cancer / Australian Ovarian Cancer Study [18]	Australia	Index (youngest affected) cases from *BRCA1*- and *BRCA2*-mutation-negative multiple-case breast and breast-ovarian families recruited though family cancer clinics from across Australia and New Zealand from 1998-present.	Identified from the electoral rolls from across Australia as part of the Australian Ovarian Cancer Study in 2002-2006.
LMBC	Leuven Multidisciplinary	Belgium	All patients diagnosed with breast cancer and	Blood donors at Gasthuisberg Hospital (200-

	Breast Centre<br>[19]		seen in the Multidisciplinary Breast Center in Leuven (Gashuisberg) since June 2007 plus retrospective collection of cases diagnosed since 2000.	2008).
MARIE	Mammary Carcinoma Risk Factor Investigation<br>[20]	Germany	Incident cases diagnosed from 2001-2005 in the study region Hamburg in Northern Germany, and from 2002-2005 in the study region Rhein-Neckar-Karlsruhe in Southern Germany.	Two controls per case were randomly drawn from population registries and frequency matched by birth year and study region to the case. Controls were recruited from 2002 to 2006.
MBCSG	Milan Breast Cancer Study Group<br>[21]	Italy	Familial and/or early onset breast cancer patients (aged 22-87) negative for mutations in *BRCA1* and *BRCA2*, ascertained at two large cancer centers in Milan from 2000-present.	Female blood donors recruited at two centres in Milan from 2004-present and 2007-present.
MCBCS	Mayo Clinic Breast Cancer Study<br>[22]	U.S.A.	Incident cases residing in 6 states (MN, WI, IA, IL, ND, SD) seen at the Mayo Clinic in Rochester, MN from 2002-2010.	Women presenting for general medical examination at the Mayo Clinic from 2002-2010; frequency matched to cases on age, ethnicity and county/state.
MCCS	Melbourne Collaborative Cohort Study<br>[23]	Australia	Incident cases from the cohort of 24,469 women, diagnosed during the follow-up from baseline (1990-1994) to 2008.	Random sample of the initial cohort.
MEC	Multiethnic Cohort<br>[24]	U.S.A.	Incident cases identified from SEER cancer registries in Los Angeles County & State registries in California & Hawaii, USA from 1993-2002. Grouped by self-reported ethnicity.	Women without cancer from the same States, recruited concurrently with cases & frequency matched to cases by age at blood-draw & self-reported ethnicity.
NBCS*	Norwegian Breast Cancer Study<br>[25]	Norway	Incidence cases from three different hospitals: Ullevål Univ. Hospital 1990-94, Norwegian Radium Hospital 1975-1986 and 1995-1998, Haukeland Univ. Hospital 1992-2001.	Women residing in Tromsø and Bergen who attended the Norwegian Breast Cancer Screening Program.
OBCS	Oulu Breast Cancer Study<br>[26]	Finland	Consecutive incident cases diagnosed at the Oulu University Hospital between 2000-2004.	Female blood donors recruited in 2002 from the same geographical region in Northern Finland.
OFBCR‡	Ontario Familial Breast Cancer Registry<br>[27]	Canada	Invasive cases aged 20-54 and a random sample aged 55-69 years identified from the Ontario Cancer Registry from 1996-1998. All	Identified by calling randomly selected residential telephone numbers in the same geographical region from 1998-2001;

			those at high genetic risk were eligible; random samples of women not meeting these criteria were also asked to participate. During 2001-2005, enrolment was limited to minority and high-risk families.	frequency matched to cases by age in 5 year categories.
ORIGO	Leiden University Medical Centre Breast Cancer Study [28,29]	Netherlands	Consecutive case patients diagnosed 1996–2006 in 2 hospitals in South–West Netherlands (Leiden & Rotterdam). No selection for family history; Rotterdam case patients selected for diagnosis aged <70. Case patients with in situ carcinomas eligible.	(1) Blood bank healthy donors from Southwest Netherlands recruited in 1996, 2000 or 2007; (2) People who married a person who was part of a family with high breast cancer risk (BRCA1/2/X). From the Southwest of the Netherlands, recruited 1990–1996; (3) Females tested at the local clinical genetics department for familial diseases, excluding familial cancer syndromes (no mutation found in gene(s) related to the disease being tested), recruited 1995–2007.
PBCS	NCI Polish Breast Cancer Study [30]	Poland	Incident cases identified through a rapid identification system in participating hospitals covering ~ 90% of all eligible cases, and cancer registries in Warsaw and Łódź covering 100% of all eligible cases (2000-2003).	Randomly selected from population lists of all residents of Poland from 2000-2003, stratified and frequency matched to cases on city and age in 5-year categories.
pKARMA	Karolinska Mammography Project for Risk Prediction Breast Cancer [31]	Sweden	Incident cases from Jan 2001 – Dec 2008 from the Stockholm/Gotland area. Identified through the Stockholm breast cancer registry.	Unmatched participants of the KARMA mammography screening study recruited between 2010 and 2011 from Southern Sweden and Stockholm.
RBCS	Rotterdam Breast Cancer Study [32]	Netherlands	Familial breast cancer patients selected from the clinical genetics center at Erasmus Medical Center between 1994-2005.	Spouses or mutation-negative siblings of heterozygous Cystic Fibrosis mutation carriers selected from the clinical genetics centre at Erasmus Medical Center between 1996-2006.
SASBAC	Singapore and Sweden Breast Cancer Study [33]	Sweden	Women diagnosed in Sweden aged 50-74 in 1993-1995.	Population-based controls frequency matched by age to the cases.
SBCS	Sheffield Breast Cancer Study [34]	U.K.	Women with breast cancer recruited in 1998-2005 at surgical outpatient clinics at the Royal Hallamshire Hospital, Sheffield.	Unselected women attending the Sheffield Mammography Screening Service in 2000-2004 with no evidence of a breast lesion.

SEARCH	Studies of Epidemiology ar Risk Factors in Cancer Heredity [35,36]	U.K.	Identified through the Eastern Cancer Registration and Information Centre: (i) prevalent cases; diagnosed 1991-1996; under 55 years of age at diagnosis; recruited 1996-2002 (ii) incident cases; diagnosed since 1996; under 70 years of age at diagnosis; recruited 1996-present.	(a) Women from the same geographic region selected from the EPIC-Norfolk cohort study, 1992-1994 (b) women attending GP practices, frequency matched to cases by age and geographic region (2003-2010) (c) women attending for breast screening as part of the NHSBSP participating in the Sisters in Breast Screening (SIBS) study
SKKDKFZ *	Städtisches Klinikum Karlsruhe Deutsches Krebsforschungszentrum Study [37]	Germany	Women diagnosed with primary *in situ* or invasive breast cancer at the Städtisches Klinikum Karlsruhe from March 1993 to July 2005. Cases were 21-93 years of age.	Controls for triple negative cases were from an unselected series of unaffected women from the same geographical region.
SZBCS	Szczecin Breast Cancer Study [38]	Poland	Prospectively ascertained cases of invasive breast cancer patients diagnosed at the Regional Oncology Hospital (2002-2003 and 2006-2007) or the University Hospital (2002-2007), both in Szczecin, West Pomerania, Poland.	Selected from a population-based study of the 1.3 million inhabitants of West Pomerania (2003-2004); matched to cases for year of birth, sex and region.
TNBCC*	Triple Negative Breast Cancer Consortium Study [39]	Various	Triple negative invasive breast cancer cases from multiple countries	Women free of breast cancer from the same geographic regions as cases
UKBGS	Breakthrough Generations Study [40]	U.K.	Cohort members who developed breast cancer or in situ breast cancer after entry into the Breakthrough Generations Study (cohort of >100,000 women followed up for breast cancer, recruited from the UK during 2003-2010).	Women who had not had breast cancer or in situ breast cancer selected by 1:1 matching to cases on date of birth, year of entry in to the study (2003-2010), source of recruitment, availability of blood sample and ethnicity.

BCAC, Breast Cancer Association Consortium; COGS, Collaborative Oncological Gene-environment Study; * CTS, NBCS and SKKDKFZ are studies in BCAC but were genotyped as part of the triple negative consortium (TNBCC). Part of GENICA was also genotyped as part of TNBCC.
†Australian site of the Breast Cancer Family Registry; ‡Ontario site of the Breast Cancer Family Registry

References:

1. Dite GS, Jenkins MA, Southey MC, Hocking JS, Giles GG, et al. (2003) Familial risks, early-onset breast cancer, and BRCA1 and BRCA2 germline mutations. J Natl Cancer Inst 95: 448-457.
2. Schmidt MK, Tollenaar RA, de Kemp SR, Broeks A, Cornelisse CJ, et al. (2007) Breast cancer survival and tumor characteristics in premenopausal women carrying the CHEK2*1100delC germline mutation. J Clin Oncol 25: 64-69.
3. Schrauder M, Frank S, Strissel PL, Lux MP, Bani MR, et al. (2008) Single nucleotide polymorphism D1853N of the ATM gene may alter the risk for breast cancer. J Cancer Res Clin Oncol 134: 873-882.
4. Fletcher O, Johnson N, Palles C, dos Santos Silva I, McCormack V, et al. (2006) Inconsistent association between the STK15 F31I genetic polymorphism and breast cancer risk. J Natl Cancer Inst 98: 1014-1018.
5. Colleran G, McInerney N, Rowan A, Barclay E, Jones AM, et al. (2010) The TGFBR1*6A/9A polymorphism is not associated with differential risk of breast cancer. Breast Cancer Res Treat 119: 437-442.
6. Yang R, Dick M, Marme F, Schneeweiss A, Langheinz A, et al. (2011) Genetic variants within miR-126 and miR-335 are not associated with breast cancer risk. Breast Cancer Res Treat 127: 549-554.
7. Villeneuve S, Fevotte J, Anger A, Truong T, Lamkarkach F, et al. (2011) Breast cancer risk by occupation and industry: analysis of the CECILE study, a population-based case-control study in France. Am J Ind Med 54: 499-509.
8. Weischer M, Bojesen SE, Tybjaerg-Hansen A, Axelsson CK, Nordestgaard BG (2007) Increased risk of breast cancer associated with CHEK2*1100delC. J Clin Oncol 25: 57-63.
9. Milne RL, Ribas G, Gonzalez-Neira A, Fagerholm R, Salas A, et al. (2006) ERCC4 associated with breast cancer risk: a two-stage case-control study using high-throughput genotyping. Cancer Res 66: 9420-9427.
10. Bernstein L, Allen M, Anton-Culver H, Deapen D, Horn-Ross PL, et al. (2002) High breast cancer incidence rates among California teachers: results from the California Teachers Study (United States). Cancer Causes Control 13: 625-635.
11. Widschwendter M, Apostolidou S, Raum E, Rothenbacher D, Fiegl H, et al. (2008) Epigenotyping in peripheral blood cell DNA and breast cancer risk: a proof of principle study. PLoS One 3: e2656.
12. Pesch B, Ko Y, Brauch H, Hamann U, Harth V, et al. (2005) Factors modifying the association between hormone-replacement therapy and breast cancer risk. Eur J Epidemiol 20: 699-711.
13. Justenhoven C, Pierl CB, Haas S, Fischer HP, Baisch C, et al. (2008) The CYP1B1_1358_GG genotype is associated with estrogen receptor-negative breast cancer. Breast Cancer Res Treat 111: 171-177.
14. Heikkinen T, Karkkainen H, Aaltonen K, Milne RL, Heikkila P, et al. (2009) The breast cancer susceptibility mutation PALB2 1592delT is associated with an aggressive tumor phenotype. Clin Cancer Res 15: 3214-3222.
15. Bogdanova NV, Antonenkova NN, Rogov YI, Karstens JH, Hillemanns P, et al. (2010) High frequency and allele-specific differences of BRCA1 founder mutations in breast cancer and ovarian cancer patients from Belarus. Clin Genet 78: 364-372.
16. Margolin S, Werelius B, Fornander T, Lindblom A (2004) BRCA1 mutations in a population-based study of breast cancer in Stockholm County. Genet Test 8: 127-132.

17. Hartikainen JM, Tuhkanen H, Kataja V, Dunning AM, Antoniou A, et al. (2005) An autosome-wide scan for linkage disequilibrium-based association in sporadic breast cancer cases in eastern Finland: three candidate regions found. Cancer Epidemiol Biomarkers Prev 14: 75-80.
18. Beesley J, Jordan SJ, Spurdle AB, Song H, Ramus SJ, et al. (2007) Association between single-nucleotide polymorphisms in hormone metabolism and DNA repair genes and epithelial ovarian cancer: results from two Australian studies and an additional validation set. Cancer Epidemiol Biomarkers Prev 16: 2557-2565.
19. De Maeyer L, Van Limbergen E, De Nys K, Moerman P, Pochet N, et al. (2008) Does estrogen receptor negative/progesterone receptor positive breast carcinoma exist? J Clin Oncol 26: 335-336; author reply 336-338.
20. Flesch-Janys D, Slanger T, Mutschelknauss E, Kropp S, Obi N, et al. (2008) Risk of different histological types of postmenopausal breast cancer by type and regimen of menopausal hormone therapy. Int J Cancer 123: 933-941.
21. Catucci I, Verderio P, Pizzamiglio S, Manoukian S, Peissel B, et al. (2009) SNPs in ultraconserved elements and familial breast cancer risk. Carcinogenesis 30: 544-545; author reply 546.
22. Olson JE, Ma CX, Pelleymounter LL, Schaid DJ, Pankratz VS, et al. (2007) A comprehensive examination of CYP19 variation and breast density. Cancer Epidemiol Biomarkers Prev 16: 623-625.
23. Giles GG, English DR (2002) The Melbourne Collaborative Cohort Study. IARC Sci Publ 156: 69-70.
24. Kolonel LN, Altshuler D, Henderson BE (2004) The multiethnic cohort study: exploring genes, lifestyle and cancer risk. Nat Rev Cancer 4: 519-527.
25. Nordgard SH, Johansen FE, Alnaes GI, Bucher E, Syvanen AC, et al. (2008) Genome-wide analysis identifies 16q deletion associated with survival, molecular subtypes, mRNA expression, and germline haplotypes in breast cancer patients. Genes Chromosomes Cancer 47: 680-696.
26. Erkko H, Xia B, Nikkila J, Schleutker J, Syrjakoski K, et al. (2007) A recurrent mutation in PALB2 in Finnish cancer families. Nature 446: 316-319.
27. John EM, Hopper JL, Beck JC, Knight JA, Neuhausen SL, et al. (2004) The Breast Cancer Family Registry: an infrastructure for cooperative multinational, interdisciplinary and translational studies of the genetic epidemiology of breast cancer. Breast Cancer Res 6: R375-389.
28. de Bock GH, Schutte M, Krol-Warmerdam EM, Seynaeve C, Blom J, et al. (2004) Tumour characteristics and prognosis of breast cancer patients carrying the germline CHEK2*1100delC variant. J Med Genet 41: 731-735.
29. Huijts PE, Vreeswijk MP, Kroeze-Jansema KH, Jacobi CE, Seynaeve C, et al. (2007) Clinical correlates of low-risk variants in FGFR2, TNRC9, MAP3K1, LSP1 and 8q24 in a Dutch cohort of incident breast cancer cases. Breast Cancer Res 9: R78.
30. Garcia-Closas M, Brinton LA, Lissowska J, Chatterjee N, Peplonska B, et al. (2006) Established breast cancer risk factors by clinically important tumour characteristics. Br J Cancer 95: 123-129.
31. Michailidou K, Hall P, Gonzalez-Neira A, Ghoussaini M, Dennis J, et al. (2013) Large-scale genotyping identifies 41 new loci associated with breast cancer risk. Nat Genet 45: 353-361.
32. Hollestelle A, Pelletier C, Hooning M, Crepin E, Schutte M, et al. (2011) Prevalence of the variant allele rs61764370 T>G in the 3'UTR of KRAS among Dutch BRCA1, BRCA2 and non-BRCA1/BRCA2 breast cancer families. Breast Cancer Res Treat 128: 79-84.
33. Wedren S, Lovmar L, Humphreys K, Magnusson C, Melhus H, et al. (2004) Oestrogen receptor alpha gene haplotype and postmenopausal breast cancer risk: a case control study. Breast Cancer Res 6: R437-449.
34. MacPherson G, Healey CS, Teare MD, Balasubramanian SP, Reed MW, et al. (2004) Association of a common variant of the CASP8 gene with reduced risk of breast cancer. J Natl Cancer Inst 96: 1866-1869.

35. Easton DF, Pooley KA, Dunning AM, Pharoah PD, Thompson D, et al. (2007) Genome-wide association study identifies novel breast cancer susceptibility loci. Nature 447: 1087-1093.
36. Lesueur F, Pharoah PD, Laing S, Ahmed S, Jordan C, et al. (2005) Allelic association of the human homologue of the mouse modifier Ptprj with breast cancer. Hum Mol Genet 14: 2349-2356.
37. Rashid MU, Jakubowska A, Justenhoven C, Harth V, Pesch B, et al. (2005) German populations with infrequent CHEK2*1100delC and minor associations with early-onset and familial breast cancer. Eur J Cancer 41: 2896-2903.
38. Jakubowska A, Jaworska K, Cybulski C, Janicka A, Szymanska-Pasternak J, et al. (2009) Do BRCA1 modifiers also affect the risk of breast cancer in non-carriers? Eur J Cancer 45: 837-842.
39. Stevens KN, Fredericksen Z, Vachon CM, Wang X, Margolin S, et al. (2012) 19p13.1 is a triple-negative-specific breast cancer susceptibility locus. Cancer Res. 72:1795-803.
40. Swerdlow AJ, Jones ME, Schoemaker MJ, Hemming J, Thomas D, et al. (2011) The Breakthrough Generations Study: design of a long-term UK cohort study to investigate breast cancer aetiology. Br J Cancer 105: 911-917.

Supplementary Table 2. Description of the PRACTICAL studies contributing to COGS.

Study Acronym	Study Name (reference)	Design, location	Ancestry	Recruitment Base	
				Cases	Controls
CAPS	Cancer of the Prostate in Sweden [1]	Case-control	European	Identified through Swedish Cancer Registry	Population controls without a diagnosis of cancer
CPCS1	Copenhagen Prostate Cancer Study 1 [2]	Case-control Denmark	European	Hospital referrals	Copenhagen General Population Study
CPCS2	Copenhagen Prostate Cancer Study 2 [3]	Case-control Denmark		Hospital referrals	Copenhagen General Population Study
EPIC	European Prospective Investigation Into Cancer and Nutrition (BPC3) [4]	Nested case-control study, Germany, Greece, Italy, Netherlands, Spain, Sweden, UK	European	Identified through linkage through record linkage with population-based cancer registries in Italy, the Netherlands, Spain, Sweden and the UK. In Germany and Greece, follow-up is active and is achieved through checks of insurance records and cancer and pathology registries as well as via self-reported questionnaires; self-reported incident cancers	Cohort participants without a diagnosis of cancer
EPIC-Norfolk	European Prospective Investigation of Cancer - Norfolk [5]	Nested case-control study	European	Identified through record linkage with population based cancer registries	Cohort participants without a diagnosis of cancer

ESTHER	Epidemiological investigations of the chances of preventing, recognizing early and optimally treating chronic diseases in an elderly population [6]	Case-control study, Germany	European	Prostate cancer cases in all hospitals in the state of Saarland, from 2001-2003	Random sample of participants from routine health check-up in Saarland, in 2000-2002
FHCRC	Fred Hutchinson Cancer Research Center [7, 8]	Population-based, case-control, ages 35-74 years at diagnosis, King County, WA, USA	European, African	Identified through the Seattle-Puget Sound SEER cancer registry	Population-based, frequency age matched (5-year groups), ascertained through random digit dialing, King County, WA, USA
IPO-Porto	Portuguese Oncology Institute, Porto	Patient series, Portugal	European	Patients treated with open radical prostatectomy at IPO-Porto	Blood donors
MAYO	Mayo Clinic Study		European	Hospital based cases	Geographically, population via Rochester Epidemiology Project
MCCS	Melbourne Collaborative Cohort Study [9]	Nested case control, Melbourne, Victoria	European	Identified by linkage to the Victorian Cancer Registry	Cohort participants without a diagnosis of cancer
MEC	Multiethnic Cohort Study [10]	Case-control in cohort, HI and CA, U.S.	Japanese	MEC	MEC
MOFFITT	The Moffitt Group [11]	Hospital based case-control	European, African	Clinic based from Moffitt Cancer Center	Moffitt Cancer Center affiliated Lifetime cancer screening center
PCMUS	Prostate Cancer study Medical University Sofia [12]	Case-control, Sofia, Bulgaria	European	Patients of Clinic of Urology, Alexandrovska University Hospital, Sofia, Bulgaria, prostate cancer histopathologically confirmed	72 patients with verified BPH and PSA<3,5; 78 healthy controls from the MMC Biobank, no history of prostate cancer
Poland	The Poland Group	Case-control	European	Men with unselected prostate cancer, diagnosed in north-	Cancer-free men from the same population, taken from the healthy

				western Poland at the University Hospital in Szczecin	adult patients of family doctors in the Szczecin region
ProMPT	Prostate cancer : Mechanisms of progression and Treatment [13]	A study to collect samples and data from subjects with and without prostate cancer	European	Subjects attending outpatient clinics in hospitals	Subjects attending outpatient clinics in hospitals
ProtecT	Prostate testing for cancer and Treatment [14]	Trial of treatment. Samples taken from subjects invited for PSA testing from the community at nine centres across United Kingdom	European	Subjects who have a proven diagnosis of prostate cancer following testing.	Identified through invitation of subjects in the community.
QLD	Retrospective Queensland Study (QLD) and the Prostate Cancer Supportive Care and Patient Outcomes Project (ProsCan) [15]	Case-control, Queensland, Australia	European	Acquired through the Queensland node of the Australian Prostate Cancer BioResource (APCB), where cases were recruited through local urologists at the time of diagnosis .	Healthy males with no personal history of prostate cancer recruited through Queensland University of Technology using the data from the Australian Electoral Commission
SCCS	Southern Community Cohort Study [16]	Case-control in cohort, Southeastern U.S.	African	SCCS	SCCS
SEARCH	Study of Epidemiology and Risk factors in Cancer Heredity [17]	Case control, East Anglia, UK	European	Men < 70 years of age registered with prostate cancer at the population-based cancer registry, Eastern Cancer Registration and Information Centre, East Anglia,	Men attending general practice in East Anglia with no known prostate cancer diagnosis, frequency matched to cases by age and geographic region

				UK	
STHLM1	Stockholm 1 [18]	Cohort	European	Identified through Swedish Cancer Registry	Cohort participants with negative prostate biopsy.
TAMPERE	Finnish Genetic Predisposition to Prostate Cancer Study [19]	Case-control, Finland	European	Identified through linkage to the Finnish Cancer Registry and patient records; and the Finnish arm of the ERSPC study	Cohort participants without a diagnosis of cancer
UKGPCS	U.K. Genetic Prostate Cancer Study and The Prostate Cancer Research Foundation Study [20]	ICR, UK	European	Cases identified through clinics at the Royal Marsden hospital and nationwide NCRN hospitals	Ken's control- 2000
ULM	Molecular Genetics of Prostate Cancer [21]	Case-control, Germany	European	Familial cases (n=292): identified through questionnaires for family history by collaborating urologists all over Germany; sporadic cases (n=311): prostatectomy series performed in the Clinic of Urology Ulm between 2000 and 2007	Age-matched controls (n=209): age-matched men without prostate cancer and negative family history collected in hospitals of Ulm; population controls (n=145): male, fully anonymized individuals from former paternity testing at the Institute of Human Genetics, Ulm
UTAH	UTAH Study [22]	Pedigree Study, Utah USA	European	Identified in the Utah Cancer Registry	NA
WUGS					

1. Zheng SL, Sun J, Wiklund F, Smith S, Stattin P, et al. (2008) Cumulative association of five genetic variants with prostate cancer. . N Engl J Med. 358:910-9.
2. Weischer M, Nordestgaard BG, Cawthon RM, Freiberg JJ, Tybjærg-Hansen A, Bojesen SE. (2013) Short telomere length, cancer survival, and cancer risk in 47102 individuals. J Natl Cancer Inst. 105:459-68.
3. Nordestgaard BG, Palmer TM, Benn M, Zacho J, Tybjaerg-Hansen A, et al. (2012) The effect of elevated body mass index on ischemic heart disease risk: causal estimates from a Mendelian randomisation approach. PLoS Med. 9:e1001212.
4. Riboli E, Hunt KJ, Slimani N, Ferrari P, Norat T, et al. (2002) European Prospective Investigation into Cancer and Nutrition (EPIC): study populations and data collection. Public Health Nutr. 5:1113-24.

5. Day N, Oakes S, Luben R, Khaw KT, Bingham S, et al. (1999) EPIC-Norfolk: study design and characteristics of the cohort. European Prospective Investigation of Cancer. Br J Cancer. 80 Suppl 1:95-103.
6. Müller H, Raum E, Rothenbacher D, Stegmaier C, Brenner H. (2009) Association of diabetes and body mass index with levels of prostate-specific antigen: implications for correction of prostate-specific antigen cutoff values? Cancer Epidemiol Biomarkers Prev. 18:1350-6.
7. Stanford JL, Wicklund KG, McKnight B, Daling JR, Brawer MK. (1999) Vasectomy and risk of prostate cancer. Cancer Epidemiol Biomarkers Prev. 8:881-6.
8. Agalliu I, Salinas CA, Hansten PD, Ostrander EA, Stanford JL. (2008) Statin use and risk of prostate cancer: results from a population-based epidemiologic study. Am J Epidemiol.168:250-60.
9. Giles GG, English DR. The Melbourne Collaborative Cohort Study. In E Riboli and R Lambert (eds) (2002) Nutrition and lifestyle: opportunities for cancer prevention. IARC Scientific Publications No 156. Lyon; International Agency for Research on Cancer, pp 69-70.
10. Kolonel LN, Henderson BE, Hankin JH, Nomura AM, Wilkens LR, et al. (2000) A multiethnic cohort in Hawaii and Los Angeles: baseline characteristics. Am J Epidemiol. 151:346-57.
11. Lin HY, Park HY, Radlein S, Mahajan NP, Sellers TA, et al. (2011) Protein expressions and genetic variations of SLC5A8 in prostate cancer risk and aggressiveness. Urology. 78:971.e1-9.
12. Eeles RA, Kote-Jarai Z, Al Olama AA, Giles GG, Guy M, et al. (2009) Identification of seven new prostate cancer susceptibility loci through a genome-wide association study. Nat Genet. 41:1116-21.
13. http://www.prostateprompt.com
14. http://www.epi.bris.ac.uk/protect)
15. Lose F, Srinivasan S, O'Mara T, Marquart L, Chambers S, et al. (2012) Genetic association of the KLK4 locus with risk of prostate cancer. PLoS One. 7:e44520.
16. Signorello LB, Hargreaves MK, Steinwandel MD, Zheng W, Cai Q, et al. (2005) Southern community cohort study: establishing a cohort to investigate health disparities. J Natl Med Assoc. 97:972-9.
17. http://ccge.medschl.cam.ac.uk/search/
18. Aly M, Wiklund F, Xu J, Isaacs WB, Eklund M, et al. (2011) Polygenic risk score improves prostate cancer risk prediction: results from the Stockholm-1 cohort study. Eur Urol. 60:21-8.
19. Laitinen VH, Wahlfors T, Saaristo L, Rantapero T, Pelttari LM, et al. (2013) HOXB13 G84E mutation in Finland: population-based analysis of prostate, breast, and colorectal cancer risk. Cancer Epidemiol Biomarkers Prev. 22(3):452-6020.
20. http://www.icr.ac.uk/research/team_leaders/Eeles_Rosalind/Eeles_Rosalind_RES/index.shtml
21. Luedeke M1, Linnert CM, Hofer MD, Surowy HM, Rinckleb AE, et al. (2009) Predisposition for TMPRSS2-ERG fusion in prostate cancer by variants in DNA repair genes. Cancer Epidemiol Biomarkers Prev. 18:3030-5.
22. Tavtigian SV1, Simard J, Teng DH, Abtin V, Baumgard M, et al. (2001) A candidate prostate cancer susceptibility gene at chromosome 17p. Nat Genet. 27:172-80.

Supplementary Table 3. Description of the OCAC studies contributing to COGS.

Study Acronym	Study Name	Location	Study Type	Recruitment Base	
				**Cases**	**Controls**
AUS	Australian Ovarian Cancer Study / Australian Cancer Study (Ovarian Cancer)	Australia	Population-based / case-control	Through major surgical treatment centres throughout Australia & and state-based cancer registries of Queensland S. Australia & W. Australia (AOCS) & state-based cancer registries of New South Wales & Victoria (ACS)	Australian Electoral Roll (~95% complete for target age-group)
BAV	Bavarian Ovarian Cancer Cases and Controls	Southeast Germany	Population-based / case-control	Cases were patients at the Gynecologic Oncology Center at the Comprehensive Cancer Center Erlangen-Nuremberg, Department of Gynecology and Obstetrics, Erlangen University Hospital.	Controls were recruited as healthy non-cancer patients by a newspaper adverstisement (i.e., volunteers
BEL	Belgian Ovarian Cancer Study	Belgium, University Hospital Leuven	Hospital-based / case-control	All cases were prospectively recruited at the University Hospital Leuven from 2007 onwards. Cases attended the Gynecologic Oncology Unit at the Leuven University Hospital.	Controls were healthy blood donors recruited from the Blood Transfusion Center in the University Hospital Leuven
DOV	Diseases of the Ovary and their Evaluation	USA: 13 counties in western Washingt on state	Population-based / case-control	Cases were identified through the Cancer Surveillance System (CSS), a population-based registry that is part of the Surveillance, Epidemiology, and End Results (SEER) program of the U.S. National Cancer Institute.	Controls were selected by RDD using the two-stage Waksberg-Mitofsky method with a clustering factor of 5 residences per sampling unit. We used a stratified sampling design that apportioned controls into 5-year age categories, 1-year calendar intervals, and two county strata (consisting of the urban three counties

					encompassing Seattle and the more rural 10 surrounding counties), according to the anticipated distribution of these characteristics among women with invasive epithelial ovarian cancer. We aimed to enroll twice as many controls as women with invasive disease.
GER	German Ovarian Cancer Study	Germany: two regions in Baden-Wurttemberg and Rhineland-Palatinate in southern Germany	Population-based / case-control	Case subjects were diagnosed by 75 years of age with either invasive ovarian cancer or borderline tumour between 1 January 1993 and 31 December 1996 and were living in the two study regions. They were identified through frequent monitoring of hospital admission, surgery schedule, and pathology records of all 26 hospitals in the study regions, with periodic checks made against pathology institutes serving these hospitals. They were either approached in the hospital before their first discharge or invited to participate in the study by letter signed by the attending physician if discharged.	Two population controls, matched by age and study region to the respective case, were selected randomly from a list of 5000 female residents for each study region obtained from the population registries and invited by letter to participate.
GRR	Gilda Radner Familial Ovarian Cancer Registry*	USA	Familial cancer / case-only	Self-referral via registry	Not applicable
HAW	Hawaii Ovarian Cancer Case-Control Study	USA: Hawaii and Southern California	Population-based / case-control	Cases were identified through the Hawaii Tumor Registry rapid case ascertainment. For studies using only epi variables, please use: Ovarian cancer cases were identified through the rapid-reporting systems of the statewide Hawaii Tumor Registry	Controls were randomly selected from Hawaii Department of Health Annual Survey of representative households that was conducted under statutory provision (participation rate close to 100%). Control pools were supplemented with women aged 65

				and the Los Angeles county Cancer Surveillance Program.	years or older through random sampling from lists obtained from Health Care Finance Administration. Controls were frequency matched to cases for age (5-year categories) and ethnicity.
HJO	Hannover-Jena Ovarian Cancer Study	Germany	Hospital-based / case-control	Cases were patients of Hannover Medical School or the Friedrich-Schiller University Jena	Controls were healthy blood donors at Hannover Medical School
HMO	Hannover-Minsk Ovarian Cancer Study	Belarus	Case-control	Cases were patients of the Byelorussian Institute for Oncology and Medical Radiology Aleksandrov N.N., Minsk, Belarus	Controls were healthy blood donors at the Byelorussian Institute for Oncology and Medical Radiology Aleksandrov N.N., Minsk, Belarus
HOC	Helsinki Ovarian Cancer Study	Helsinki, Finland	Case-control	Cases were patients treated for invasive epithelial ovarian carcinoma at Helsinki University Central Hospital 1989-1998 and the blood samples were collected in routine follow-up visits to the clinic during 1998 from those patients who were still alive. Additional samples were prospectively collected from patients treated for ovarian cancer at Helsinki University Central Hospital Department of Obstetrics and Gynecology between 1998-2006.	Controls were healthy female blood donors at Finnish Red Cross Blood Transfusion Service
HOP	Hormones and Ovarian Cancer Prediction	USA: Western PA, Northeastern OH, Western NY	Population-based / case-control	Recruitment goal was all incident incident cases in the three catchment areas, aged 25+, with primary epithelial ovarian cancer, peritoneal cancer, cancer of the fallopian tube and/or endometrial cancer diagnosed within 9 months of recruitment. Cases [illegible] identified through a variety of sources including physician offices, cancer	Controls were ascertained by the Unive[illegible] and Social Research using random digi[illegible] matched to cases by age (5 year interva[illegible] exchange.

				registries and pathology databases. Roberta: In the absence of a SEER registry, a study can never be fully "population based." We searched hospital registries and used active surveillance of practices. So we tried to replicate SEER but without the SEER infrastructure.	
HSK	Dr. Horst Schmidt Kliniken*	Germany	Case-only	Patients were referred to our tertiary GO unit due to unknown ovarian masses by the gynecologist, or if incomplete staging or incomplete (non-optimal) debulking was accomplished in other hospitals. Moreover, some patients were referred in a later course of the disease for second opinion, or surgery for recurrent ovarian cancer. All these patients were asked for DNA sampling	Not applicable
LAX	Women's Cancer Research Institute - Cedars-Sinai Medical Center*	USA: Southern California	Case-only	Cases were identified through the Women's Cancer Program Biorepository. Participants recruited include patients scheduled for breast and gynecologic surgical procedures at CSMC or Olive View Medical Center, patients seen during clinical visits (gynonc, breast, or genetics) at CSMC with a family history of cancer, or patients with a family history of cancer who wanted to participate in research (but not necessarily seen at CSMC).	Not applicable
MAL	Danish Malignant Ovarian Tumor Study	Denmark	Population-based / case-control	From December 1, 1994 to May 30, 1999 women aged 35-79 years who	Controls from the general female population, 35 to 79 years of age in the

				were scheduled for an explorative laparotomy or laparoscopy because of suspicion of an ovarian tumor were requested to participate in the study with blood and tissue samples and a personal interview. The women were recruited from 16 gynecological departments in Denmark (municipalities of Copenhagen and Frederiksberg as well as the counties of Copenhagen, Frederiksberg, Roskilde, Western Zealand, Funen, Southern Jutland, and Northern Jutland).	study area, were drawn by means of the computerized Civil Registration System (all inhabitants in Denmark have a unique personal identification number, which is registered in the Civil Registration System). Controls were recruited simultaneously with the cases, and from the area which generated the cases. They were frequency-matched in 5-year intervals by using the age distribution of women with ovarian cancer (1987-1992) registered in the Danish Cancer Registry.
MAY	Mayo Clinic Ovarian Cancer Case Control Study	USA: North Central (MN, SD, ND, IL, IA, WI)	Clinic-based / case-control	Cases attending Mayo Clinic Division of Surgical Gynecology and Mayo Clinic Division of Medical Oncology residing in a six-state surrounding region.	Women from the same six-state region seen at Mayo Clinic's Department of Family Medicine and the Department of General Internal Medicine for general medical exams.
MCC	Melbourne Collaborative Cohort Study	Melbourn e, Australia	Cohort / Nested case-control	Cases are identified through regular linkage of the MCCS cohort to the Victorian Cancer Registry, that is the cancer registry where the cohort resides, and National Cancer Statistics (Australia). The original MCCS cohort participants were volunteers recruited through media campaigns and advertising. The electoral rolls were only one of the sources used to identify potential participatns. The cohort was deliberately enriched with migrants from Italy and Greece in order to recruit a sample with an increased range of dietary exposure.	Controls are women randomly selected from MCCS participants. Women are eligible to be selected as controls if they do not have a diagnosis of ovarian cancer at the time of diagnosis of the matching case.<br>Controls are matched to cases on a number of criteria.

MDA	MD Anderson Ovarian Cancer Study	USA: Texas	Hospital-based / case-control	Ovarian cancer cases were recruited from MD Anderson Cancer Center. All women were newly diagnosed, histologically confirmed, and previously untreated. There were no restrictions on recruitment in terms of age, ethnicity, or clinical stage of disease.	Controls were healthy women without prior history of cancer (except non-melanoma skin cancer) and identified from a large pool of control subjects enrolled in on-going case-control studies of cancer. Controls subjects were individuals seeing a physician for routine health checkups or addressing health concerns at the Kelsey–Seybold Clinic. Controls were matched to cases by age (±5 years) and ethnicity.
MSK	Memorial Sloan Kettering Cancer Center Gynecology Tissue Bank	USA: New York City	Case-control	Ovarian cancer cases are derived from women with presumed ovarian cancer who are approached for participation at the time that they sign informed consent for surgery. Cases are also collected from women receiving chemotherapy in the outpatient clinics. Cases include only women with pathologically confirmed invasive epithelial ovarian cancer. Cases are classified according to the staging system of the International Federation of Gynecology and Obstetrics (FIGO). Samples are collected from all ovarian cancer patients including primary, recurrent and neoadjuvant cases. This ascertainment continues at present.	Ovarian cancer controls include healthy women with no known history of malignancy. Controls are recruited from outpatient clinics as spouses of prostate cancer patients or unrelated companions of breast cancer patients. These controls are individually approached by research study assistants in the waiting area of the clinics. All controls were women with no personal history of breast or ovarian cancer or such history in a first degree relative and no history of oophorectomy. The control ascertainment continued until 2005 and is now complete.
NCO	North Carolina Ovarian Cancer Study	USA: Central and eastern NC (48 counties)	Population-based / case-control	Cases were identified through the North Carolina Central Cancer Registry by using rapid case ascertainment in a 48 county region of Pathology reports for ovarian cancer cases were forwarded to the Central Cancer Registry and then to the study	Controls from the same 48-county region were identified by using random digit dialing and were frequency matched to cases by age (5-year categories) and race (African American/non–African American).

				office within 2 months of diagnosis.	
NEC	New England Case-Control Study	USA: NH and Eastern MA	Population-based / case-control	Cases were identified through statewide cancer registries and hospital tumor boards in eastern Massachsetts and New Hampshire.	Controls were identified from same regions using a combination of random digit dailing, drivers' license lists, and town resident lists (called townbooks). Controls were frequency groups and state of residence.
NHS	Nurses' Health Study	USA	Population-based / nested case-control	Cases are identified by self-report on biennual questionnaires or through report of deaths by family or the postal service; the NDI is also searched on an annual basis	Controls are identified from the subset blood samples in 1990 (nested case control study). Controls must be alive of case diagnosis (incidence density matching) and are matched to cases on age, menopausal status at blood draw and diagnosis, hormone use at blood draw, fasting status, and time of day and date of blood draw.
NJO	New Jersey Ovarian Cancer Study	USA: New Jersey (6 counties)	Case-control	Newly diagnosed, pathologically confirmed cases of invasive epithelial ovarian cancer were identified through in six counties in New Jersey implemented by NJ State Cancer Registry.	Methods of ascertaining controls included random digit dialling for women under 65 years of age, random selection from the Center for Medicare and Medicaid Services (CMS) lists for women older than 65, and area sampling for women older than 55 years.
NOR	University of Bergen Norway Study	Norway	Case-control	All patients treated for suspected gynecologic cancer from one region of Norway	Control were drawn from healthy female blood donors with known age, and no known previous cancer.
NTH	Nijmegen Polygene Study & Nijmegen Biomedical Study	Eastern part of Netherlands	Case-control	Women diagnosed with ovarian cancer between 1989 and 2006 and still alive in 2008, identified from the population-based cancer registry of the Centre East in the Mid-Eastern part of the Netherlands	The controls were selected from the Nijmegen Biomedical Study (NBS). The NBS is a population-based survey performed in 2002-2003 by the Radboud University Nijmegen Medica Centre. This survey was based on an age-stratified random sample of the population of Nijmegen. Life style

					information, family history of cancer, reproductive and medical history as well as blood samples were available from a group of 6,700 individuals (males + females).
ORE	Oregon Ovarian Cancer Registry*	USA: Portland, Oregon	Case-only	Registry and tissue repository of patients with ovarian cancer (or at risk for ovarian cancer and control case undergoing surgery for benign gyneologic conditions that aren't included in OCAC). Focus of the current research is on Fanconi DNA repair genes and proteins.	Not Applicable
OVA	Ovarian Cancer in Alberta and British Columbia Study	Alberta and British Columbia, Canada	Case-control	Cases were identified through the population-based provincial cancer registries in both provinces. Pathology reports for ovarian cancer cases are forwarded to the Study Coordinators as they are received at the registries.	Controls come from two sources: 1) randomly identified from provincial health rosters in Alberta and BC (in the single-payer health care system in Canada all persons receiving health care in the province are included in the provincial health rosters); 2) a province-wide breast cancer screening program (BC).
POC	Poland Ovarian Cancer Study†	Poland: Szczecin, Poznan, Opole, Rzeszow	Case-control	Cases were identified through pathology departments of Szczecin, and pathology departments of main oncology hospitals of Poznan, Opole and Rzeszów.	Controls from the same regions were randomly selected from registries of family doctors and were matched to cases by age (1-year categories)
POL	NCI Ovarian Case-Control Study in Poland†	Poland, Warsaw, Lodz	Population-based / case-control	Rapid ascertainment at the hospitals was confirmed through cancer registries for the area.	Controls from the same regions were identified through the Polish Electronic Resident Registry and matched to cases by age and site
PVD	Danish Pelvic Mass Study*	Denmark	Case-only	Case-only	Not Applicable
RMH	Royal Marsden Hospital Case Series*	UK: London	Hospital-based / case-only	All cases of epithelia ovarian cancer diagnosed under 70 years of age	Not Applicable

				presenting at RMH and at other participating hospitals.	
SEA	UK Studies of Epidemiology and Risk Factors in Cancer Heredity (SEARCH) Ovarian Cancer Study	UK: East Anglia and West Midlands	Population-based / case-control	Cases were identified through the East Anglia and West Midlands Cancer Registry, a regional cancer registry.	Controls have been randomly selected from the EPIC-Norfolk component of the European Prospective Investigation of Cancer (EPIC), a prospective study of diet and cancer. The EPIC –Norfolk cohort comprises 25,000 individuals resident in Norfolk (East Anglia), aged 45-74 years at first interview in 1990. Some controls are from a population based controls series from East Anglia, UK. Individuals aged 25-69 will be identified through up to ten general practices in East Anglia. We calculated the proportions of cancer patients in each age band (25-29, 30-34, 35-39, etc.), and then recruited controls in the same proportions.
SOC	Southampton Ovarian Cancer Study*	UK: Wessex region	Hospital-based / case-only	SOC cases represent a sequential series of patients undergoing primary surgery for epithelial ovarian cancer at hospitals in the Wessex region of southern England.	Not Applicable
SRO	Scottish Randomised Trial in Ovarian Cancer*	Coordinated through clinical trials unit, Glasgow UK from patients recruited worldwide	Case-only from clin	Cases were patients enrolled on the Phase III SCOTROC1 trial	Not Applicable

STA	Familial Registry for Ovarian Cancer and Genetic Epidemiology of Ovarian Cancer	USA: San Francisco bay (6 counties)	Population-based / case-control	Cases were identified via rapid case ascertainment through the Greater Bay Area Cancer Registry operated by the Cancer Prevention Institute of California (formerly the Northern California Cancer Center)) as part of the National Cancer Institute's Surveillance, Epidemiology, End Results Program.	Control women living in the six Bay Area counties were identified through random-digit dial and were frequency-matched to cases by five-year age groups and by race/ethnicity
TBO	Tampa Bay Ovarian Cancer Study	USA: Florida	Population-based / case-control	Cases were identified through a rapid case-ascertainment mechanism that included 7 regional gynecologic and medical oncologists who treat 85% of women with ovarian carcinoma in the area to allow preoperative enrollment and specimen collection from all women suspected to have ovarian cancer in the Tampa Bay, Florida metropolitan area.	Controls were identified from the Lifetime Cancer Screening center (same geographic regions as cases) for frequency matching by age and race.
TOR	Toronto Ovarian Cancer Study	Canada: Province of Ontario	Population-based / case-control	FOTS: Case subjects were identified by use of records supplied by the Ontario Cancer Registry, covering all hospitals in the province. Pathology reports were reviewed for all cases. HW: controls only so not applicable	All >1000 controls with DNA are from HW. Population-based collection. FOTS: Cases identified sisters, sisters-in-law and female cousins and cousins-in-law resident in Ontario and generally within +/- 5 years of age as potential controls. Controls were sought in the following order: sister-in-law, cousin-in-law, cousin, sister; Only 200 FOTS controls were collected. Epi data for the 1000 HW controls and the 200 FOTS controls is in the OCAC
UCI	UC Irvine Ovarian Cancer Study	USA: Southern	Population-based / case-control	Cases were identified through the California Cancer Registry by using	Controls were identified by using random digit dialing from Orange and

		California (Orange, San Diego, and Imperial counties)		rapid case ascertainment in Orange and San Diego counties. Pathology reports for ovarian cancer cases were forwarded to study coordinators.	San Diego counties, frequency matched on race/ethnicity and 5 year age categories.
UKO	UK Ovarian Cancer Population Study	UK: England, Wales, Northern Ireland	Population-based / case-control	Cases attending ten major Gynaecological Oncology NHS centers in England, Wales and Northern Ireland from 2006 to 2010.	Controls were apparently healthy postmenopausal women aged 50 to 74 from the general population participating in the United Kingdom Collaborative Trial of Ovarian Cancer Screening (UKCTOCS). All women followed up for cancers through the Office of National Statistics for cancer registration.
UKR	UK Familial Ovarian Cancer Registry[*]	UK: National	Case-only / familial register	Eligible familes were those with at least two confirmed cases of invasive EOC in first or second degree relatives	Not Applicable
USC	Los Angeles County Case-Control Studies of Ovarian Cancer	USA: Southern California (Los Angeles County)	Population-based / case-control	Cases were identified through the Los Angeles County Cancer Surveillance Program (part of SEER) by rapid case ascertainment.	Individually matched neighbourhood controls or for cases >65 for whom a neighbourhood control could not be identified, we also used a random sample from Health Care Financing Administration
WOC	Warsaw Ovarian Cancer Study[†]	Poland: Warsaw and central Poland	Case-control	Cases were identified in two Warsaw hospitals. The criteria of inclusion into the present series were: 1) either the patients had fitted the criteria of inclusion into a study on prognosis and tumor response to chemotherapy and/or 2) they were on the list of frozen or paraffin -embedded tumors in our tissue bank; 3) a final (limiting) condition was the availability of blood from those	Controls were collected in a short period of time: 800 samples within three months of the 2009 at blood donation center in Warsaw and 180 samples among employees of the hospital; there should not be any repetitive cases, since blood donors are advised not to come twice within 3 months and we also had asked the staff who collected blood not to include repetitive cases. Our control

				patients.	group is women only.

[*] Case only study. For our analyses, GRR was merged with HOP, HSK with GER, LAX with USC, ORE with DOV, PVD with MAL, and RMH, SOC, SRO, and UKR with UKO.
[†] The three Polish studies were combined in our analyses.
[ǂ] Numbers do not equal total number of invasive cases because some cases were not classified as one of the four histological subtypes.
[**] Total number of controls included an additional 6116 controls from the ovarian cancer GWAS in the U.K.

Supplementary Figure 1. Cluster Plots of the ten variants included in this study.

PALB2 c.1592delT (p.Leu531Cysfs)

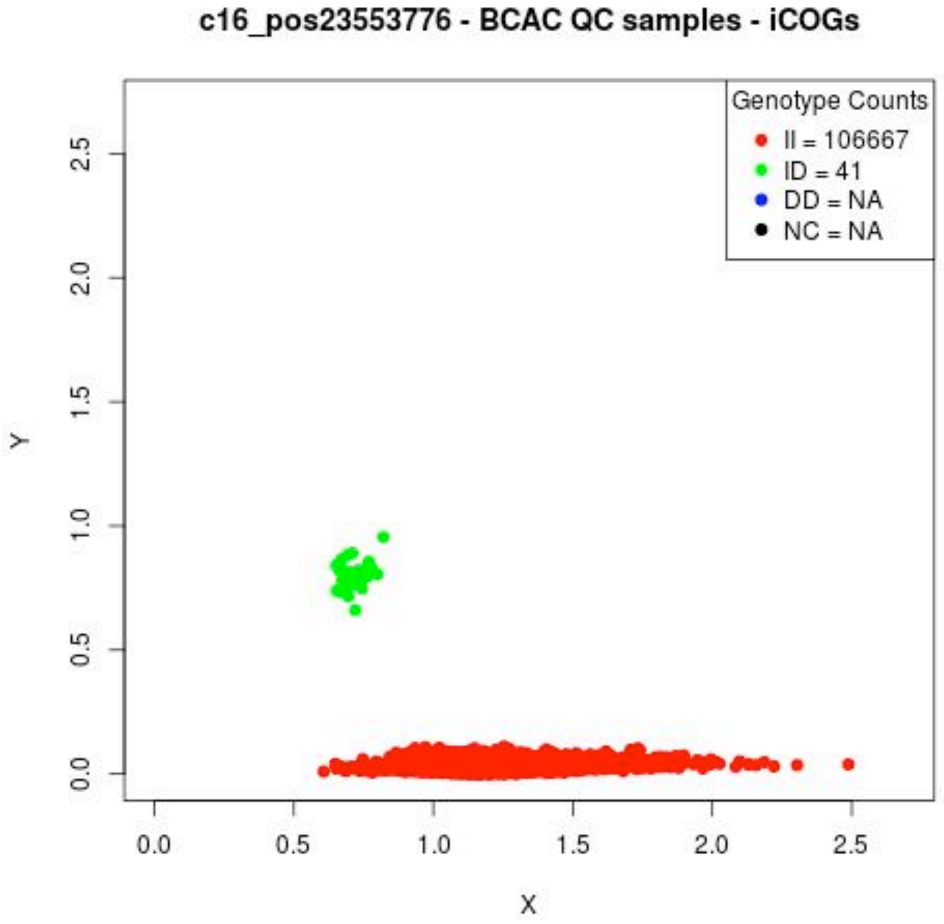

PALB2 c.2816T>G (p.Leu939Trp)

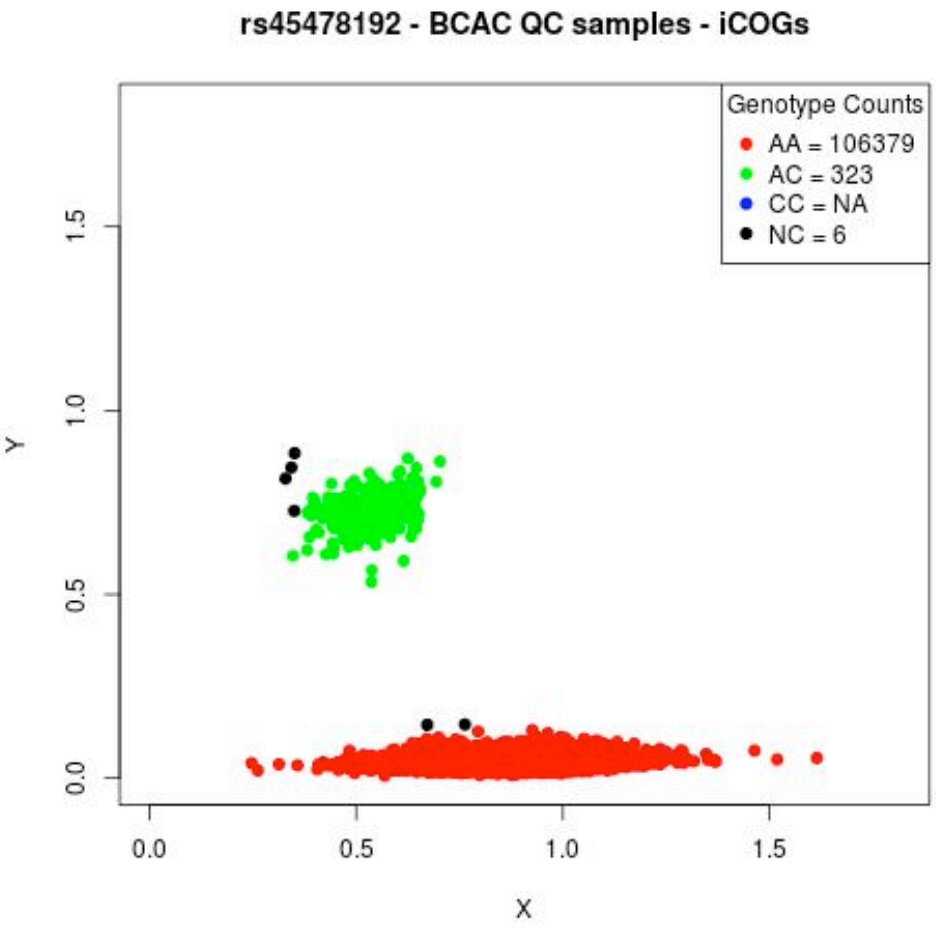

PALB2 c.3113G>A (p.Trp1038Ter)

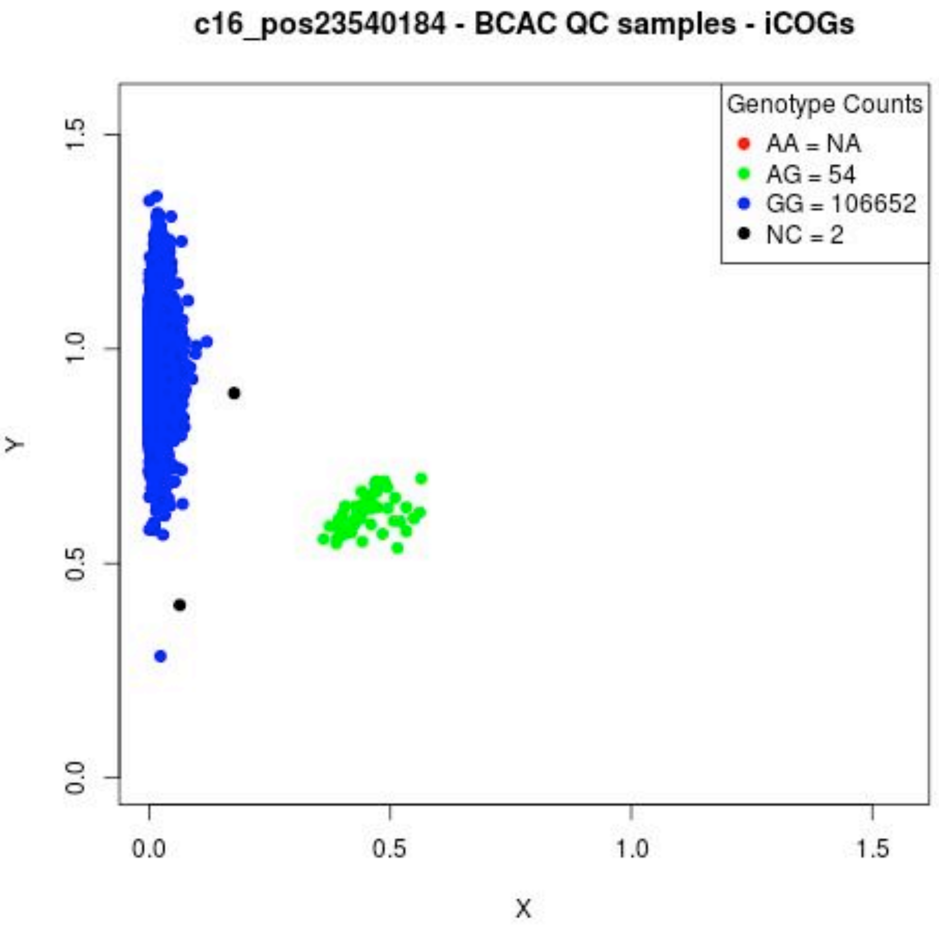

CHEK2 c.349A>G (p.Arg117Gly)

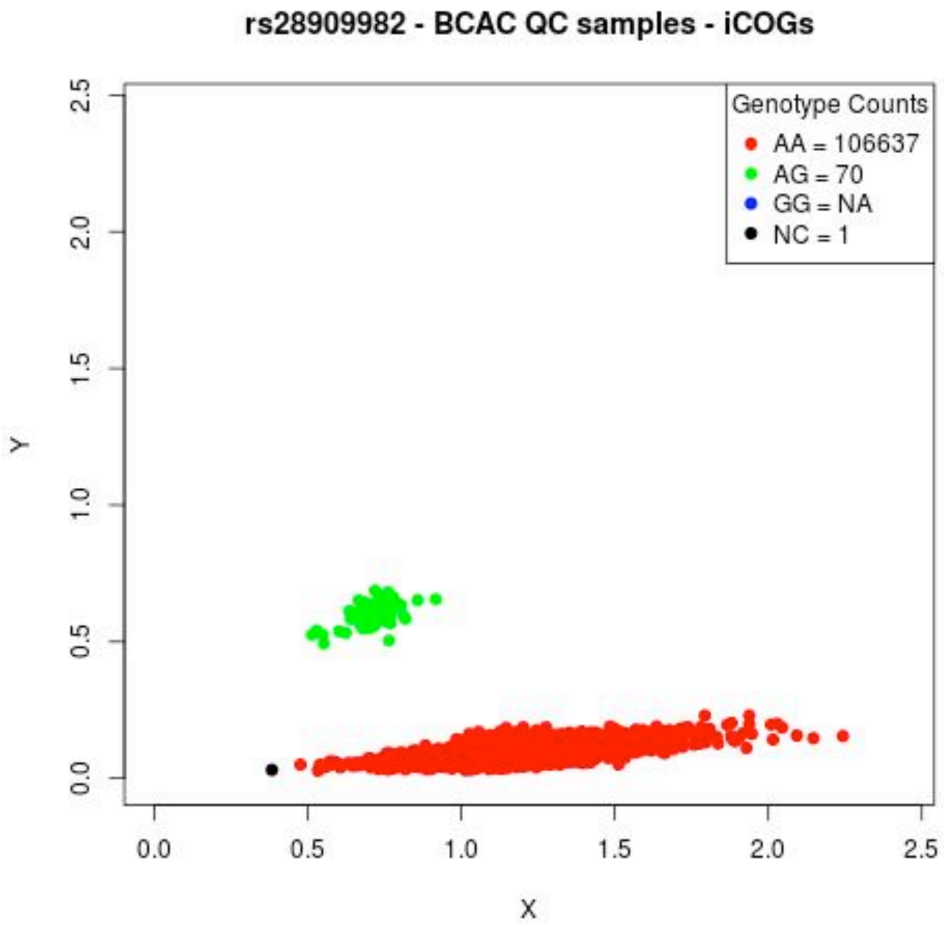

CHEK2 c.538C>T (p.Arg180Cys)

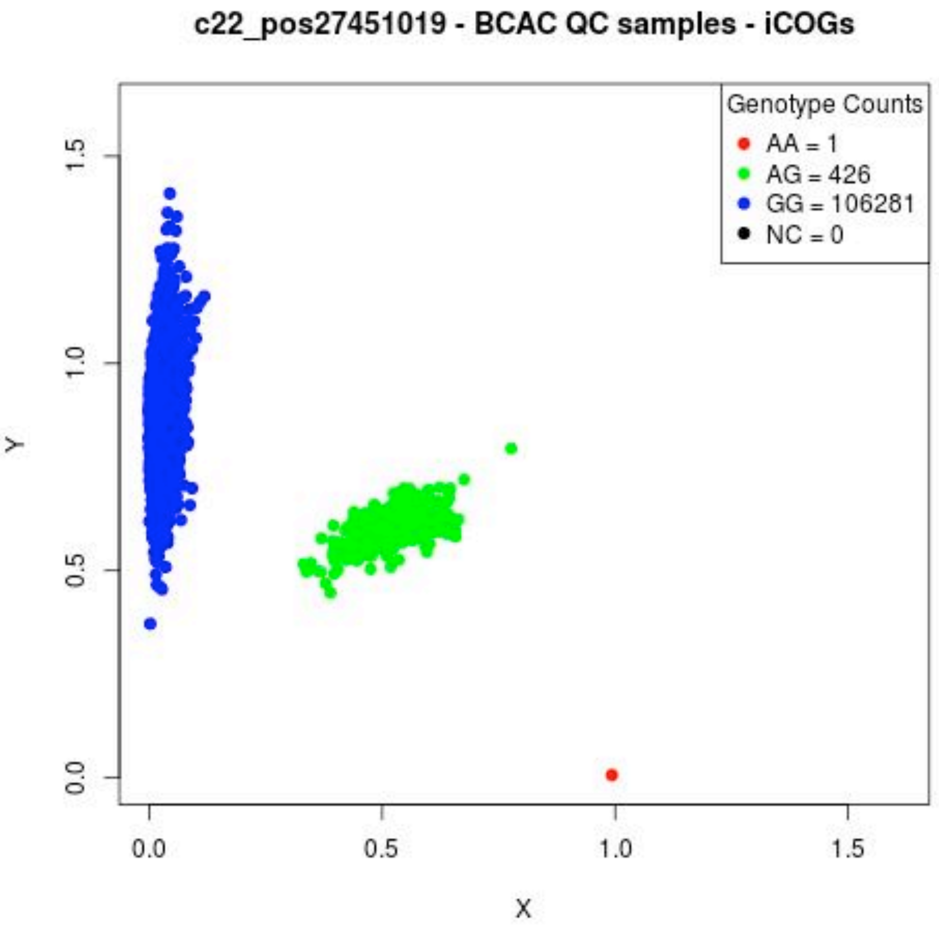

CHEK2 c.715G>A (p.Glu239Lys)

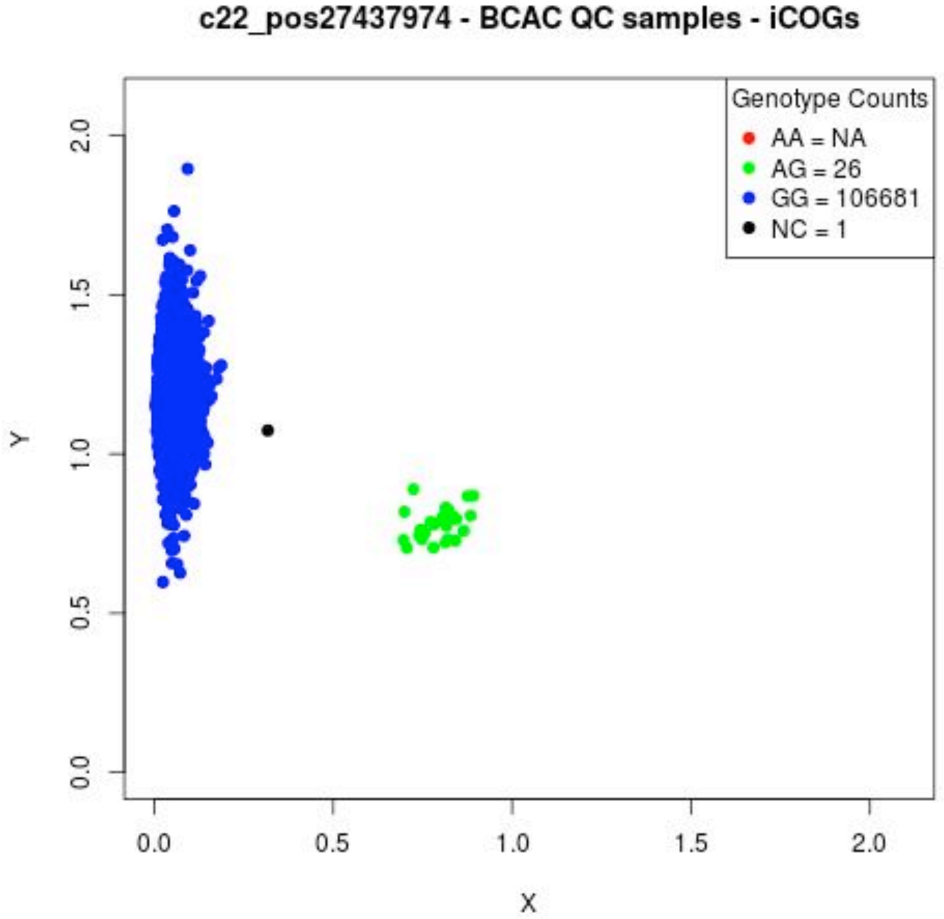

CHEK2 c.1036C>T (p.Arg346Cys)

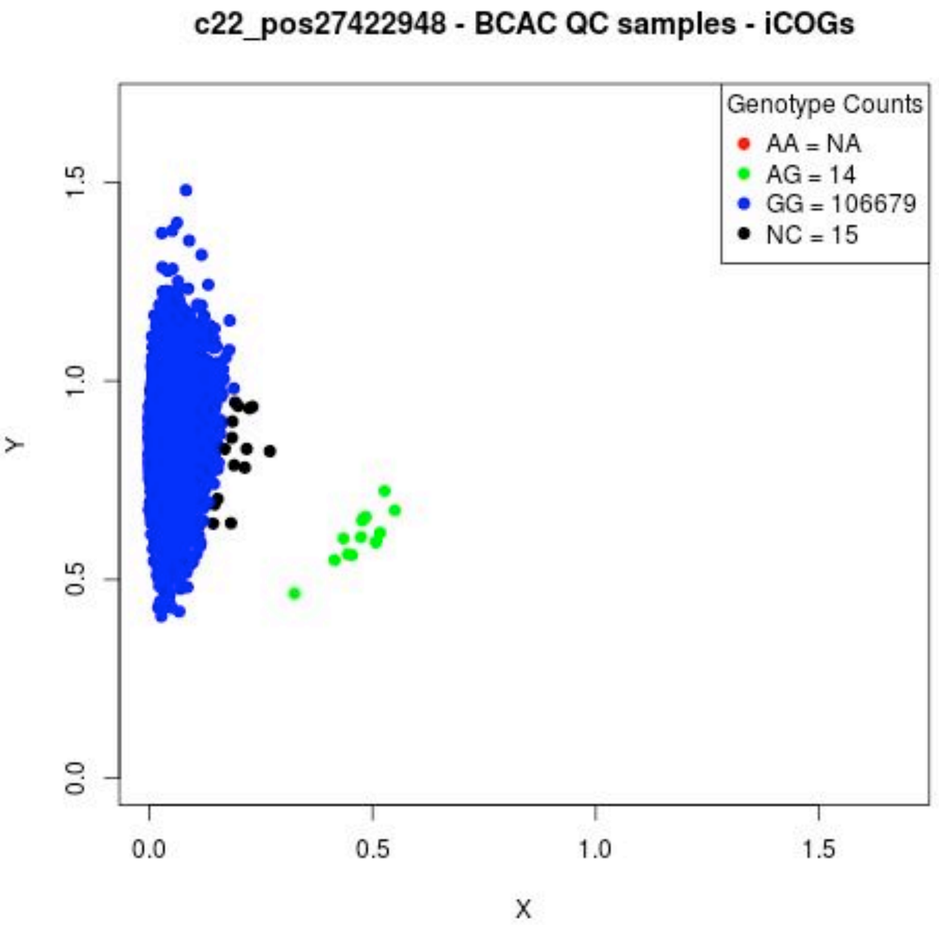

CHEK2 c.1312G>T (p.Asp438Tyr)

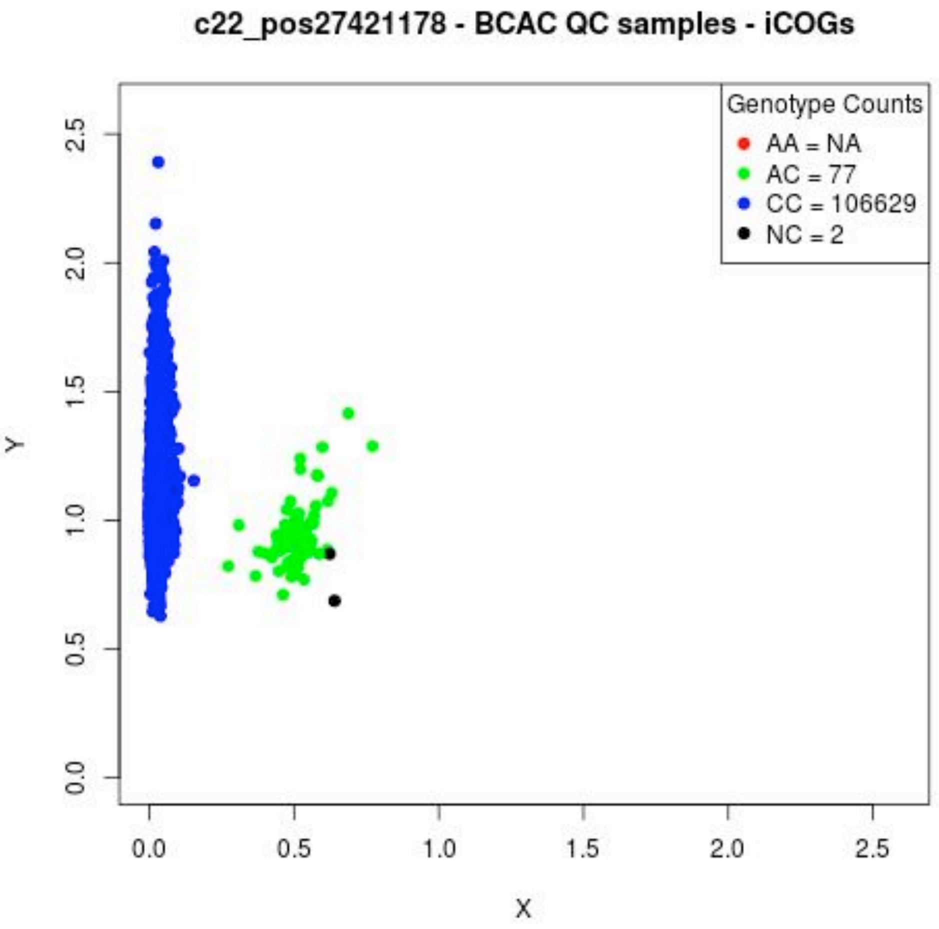

CHEK2 c.1343G>T (p.Ile448Ser)

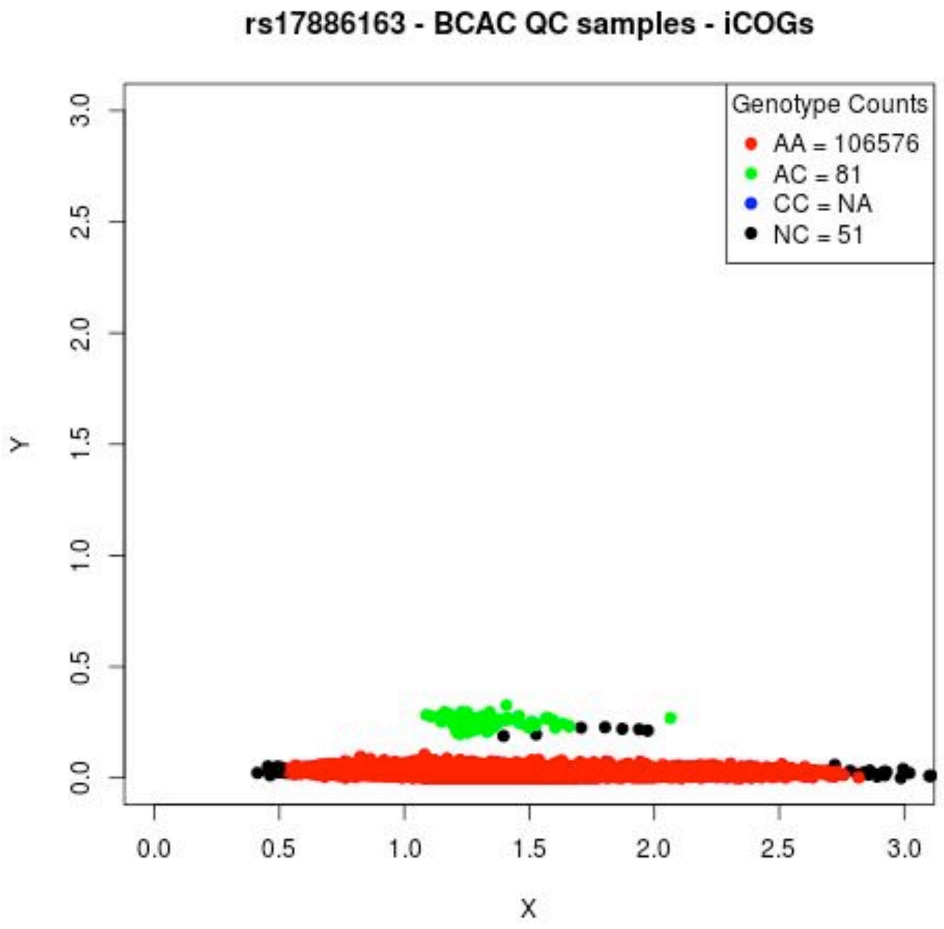

ATM c.7271T>G (P.Val2424Gly)

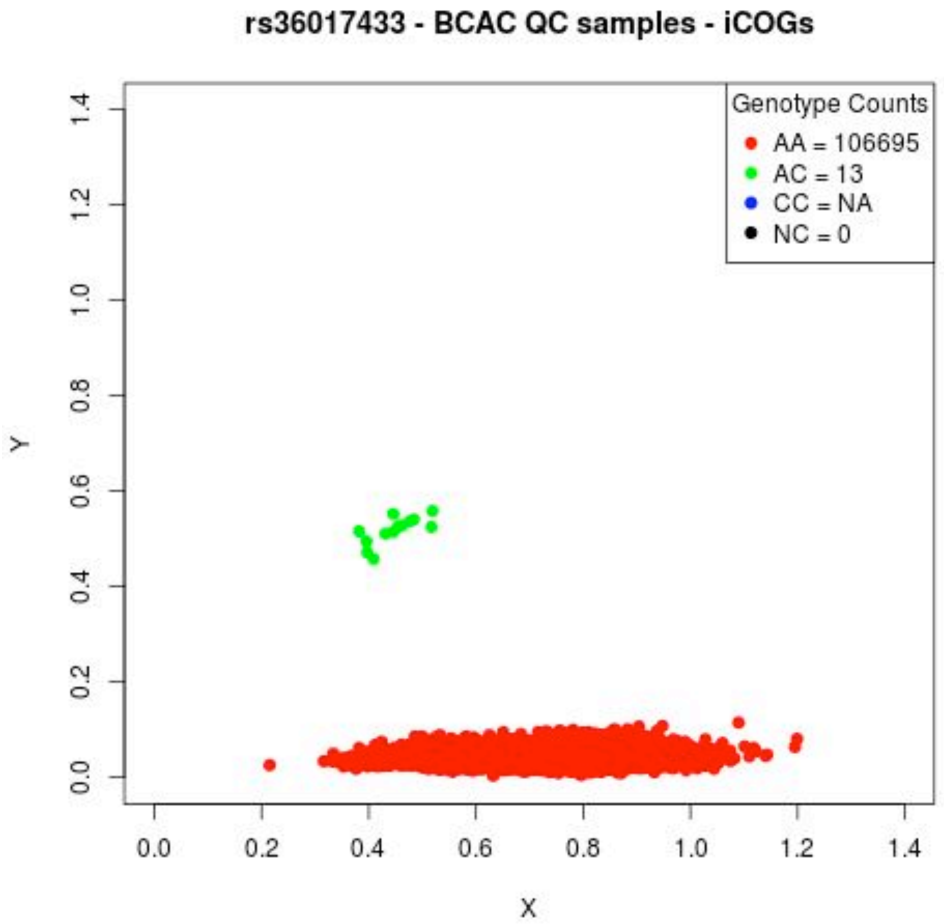

Supplement: Supplementary data [file jmedgenet-2016-103839supp.pdf]
