# Supplementary material for: PALB2, CHEK2 and ATM rare variants and cancer risk: data from COGS
Source: J Med Genet. 2016 Sep 5;53(12):800–11. doi: 10.1136/jmedgenet-2016-103839 (PMC5200636; doi:10.1136/jmedgenet-2016-103839)
Supplement: Supplementary Financial Support [file jmedgenet-2016-103839supp_financial_support.pdf]

Additional Financial Support

For Breast Cancer Association Consortium (BCAC) studies:

The **UK2** GWAS was funded by Wellcome Trust and Cancer Research UK. It included samples collected through the FBCS study which is funded by Cancer Research UK [C8620/A8372]. The WTCCC was funded by the Wellcome Trust. The **EBCG-GWAS** was supported by National Institute of Health Grants U01CA122171, RC1CA145506, R01CA094069, and U19CA148065. The Breast Cancer Family Registry (**BCFR**) is supported by the National Cancer Institute, National Institutes of Health under RFA-CA-06-503 and through cooperative agreements with members of the BCFR and Principal Investigators, including Cancer Care Ontario (U01 CA69467), Cancer Prevention Institute of California (CPIC) (U01 CA69417), and University of Melbourne (U01 CA69638). Samples from the CPIC were processed and distributed by the Coriell Institute for Medical Research. The Northern California site of the Breast Cancer Family Registry [**BCFR (NCA)**] was supported by grant UM1 CA164920 from the National Cancer Institute (USA). The content of this article does not necessarily reflect the views or policies of the National Cancer Institute or any of the collaborating centers in the BCFR, nor does mention of trade names, commercial products, or organizations imply endorsement by the U.S. Government or the BCFR. The **BPC3** was supported by the US National Institutes of Health, National Cancer Institute under cooperative agreements U01-CA98233 (NHS, NHSII, WHS), U01-CA98710 (CPS2), U01-CA98216 (EPIC), U01-CA98758 (MEC) and Intramural Research Program of NIH/National Cancer Institute, Division of Cancer Epidemiology and Genetics (PLCO). The authors thank Drs Christine Berg and Philip Prorok, Division of Cancer Prevention, NCI, the screening center investigators and staff of the PLCO Cancer Screening Trial, Mr Thomas Riley and staff at Information Management Services, Inc., and Ms Barbara O'Brien and staff at Westat, Inc. for their contributions to the PLCO Cancer Screening Trial. We would like to thank the participants and staff of the NHS and NHSII for their valuable contributions as well as the following state cancer registries for their help: AL, AZ, AR, CA, CO, CT, DE, FL, GA, ID, IL, IN, IA, KY, LA, ME, MD, MA, MI, NE, NH, NJ, NY, NC, ND, OH, OK, OR, PA, RI, SC, TN, TX, VA, WA, WY. The authors assume full responsibility for analyses and interpretation of these data. The Australian Breast Cancer Family Study (**ABCFS**) was supported by grant UM1 CA164920 from the National Cancer Institute (USA). The ABCFS was also supported by the National Health and Medical Research Council of Australia, the New South Wales Cancer Council, the Victorian Health Promotion Foundation (Australia) and the Victorian Breast Cancer Research Consortium. J.L.H. is a National Health and Medical Research Council (NHMRC) Australia Fellow and a Victorian Breast Cancer Research Consortium Group Leader. M.C.S. is a NHMRC Senior Research Fellow and a Victorian Breast Cancer Research Consortium Group Leader. We thank Maggie Angelakos, Judi Maskiell and Gillian Dite. The **ABCS** was supported by the Dutch Cancer Society [grants NKI 2007-3839; 2009 4363]; BBMRI-NL, which is a Research Infrastructure financed by the Dutch government (NWO 184.021.007); and the Dutch National Genomics Initiative.We thank Sten Cornelissen, Richard van Hien, Linde Braaf, Frans Hogervorst, Senno Verhoef, Laura van 't Veer, Emiel Rutgers, C Ellen van der Schoot and Femke Atsma. The **ACP** study is funded by the Breast Cancer Research Trust, UK. The **ACP** study wishes to thank the participants in the Thai Breast Cancer study. Special Thanks also go to the Thai Ministry of Public Health (MOPH), doctors and nurses who helped with the data collection process. The study would like to thank Dr Prat Boonyawongviroj, the former Permanent Secretary of MOPH and Dr Pornthep Siriwanarungsan, the Department of Health Director-General who have supported the study throughout. The work of the **BBCC** was partly funded by ELAN-Fond of the University Hospital of Erlangen. The **BBCS** is funded by Cancer Research UK and Breakthrough Breast Cancer and acknowledges NHS funding to the NIHR Biomedical Research Centre, and the National Cancer Research Network (NCRN). The BBCS GWAS received funding from The Institut National de Cancer. We thank Eileen Williams, Elaine Ryder-Mills and Kara Sargus. **BIGGS. ES** is supported by NIHR Comprehensive Biomedical Research Centre, Guy's & St. Thomas' NHS Foundation Trust in partnership with King's College London, United Kingdom. IT is supported by the Oxford Biomedical Research Centre. We thank Niall McInerney, Gabrielle Colleran, Andrew Rowan and Angela Jones. The **BSUCH** study was supported by the Dietmar-Hopp Foundation, the Helmholtz Society and the German Cancer Research Center (DKFZ). We thank Peter

Bugert and the Medical Faculty Mannheim. The **CECILE** study was funded by Fondation de France, Institut National du Cancer (INCa), Ligue Nationale contre le Cancer, Ligue contre le Cancer Grand Ouest, Agence Nationale de Sécurité Sanitaire (ANSES) and Agence Nationale de la Recherche (ANR). The CECILE study would like to thank Pierre Kerbrat (Centre Eugène Marquis, Rennes) and Patrick Arveux (Centre Georges-François Leclerc, Dijon). **CGEMS.** The Nurses' Health Studies are supported by NIH grants CA 65725, CA87969, CA49449, CA67262, CA50385 and 5UO1CA098233.The **CTS** was initially supported by the California Breast Cancer Act of 1993 and the California Breast Cancer Research Fund (contract 97-10500) and is currently funded through the National Institutes of Health (R01 CA77398). Collection of cancer incidence data was supported by the California Department of Public Health as part of the statewide cancer reporting program mandated by California Health and Safety Code Section 103885. HAC receives support from the Lon V Smith Foundation (LVS39420). The CTS Steering Committee includes Leslie Bernstein, Susan Neuhausen, James Lacey, Sophia Wang, Huiyan Ma, Yani Lu, and Jessica Clague DeHart at the Beckman Research Institute of City of Hope, Dennis Deapen, Rich Pinder, Eunjung Lee, and Fred Schumacher at the University of Southern California, Pam Horn-Ross, Peggy Reynolds, Christina Clarke Dur and David Nelson at the Cancer Prevention Institute of California, and Hoda Anton-Culver, Argyrios Ziogas, and Hannah Park at the University of California Irvine. The **CGPS** was supported by the Chief Physician Johan Boserup and Lise Boserup Fund, the Danish Medical Research Council and Herlev Hospital. The CGPS thanks the staff and participants of the Copenhagen General Population Study, and Dorthe Uldall Andersen, Maria Birna Arnadottir, Anne Bank, Dorthe Kjeldgård Hansen for excellent technical assistance. The **CNIO-BCS** was supported by the Genome Spain Foundation, the Red Temática de Investigación Cooperativa en Cáncer and grants from the Asociación Española Contra el Cáncer and the Fondo de Investigación Sanitario (PI11/00923 and PI081120). The Human Genotyping-CEGEN Unit (CNIO) is supported by the Instituto de Salud Carlos III. We thank Guillermo Pita, Charo Alonso, Daniel Herrero, Nuria Álvarez, Pilar Zamora, Primitiva Menendez and the Human Genotyping-CEGEN Unit (CNIO). The **DFBBCS** GWAS was funded by The Netherlands Organisation for Scientific Research (NWO) as part of a ZonMw/VIDI grant number 91756341. We thank Margreet Ausems, Christi van Asperen, Senno Verhoef, and Rogier van Oldenburg for providing samples from their Clinical Genetic centers. The **ESTHER** study was supported by a grant from the Baden Württemberg Ministry of Science, Research and Arts. Additional cases were recruited in the context of the VERDI study, which was supported by a grant from the German Cancer Aid (Deutsche Krebshilfe). We thank Hartwig Ziegler, Sonja Wolf and Volker Hermann. The **FHCRC** Studies were supported by NIH grants CA056678, CA082664 and CA092579. The **GC-HBOC** was supported by Deutsche Krebshilfe (107 352). We thank Heide Hellebrand, Stefanie Engert, Prof. Dr. Norbert Arnold, Dr. Sabine Preissler-Adams, Dr. Monika Mareeva-Varon, Dr. Dieter Niederacher, Prof. Dr. Brigitte Schlegelberger and Dr. Clemens Mül. The **GENICA Network** was funded by the Federal Ministry of Education and Research (BMBF) Germany grants 01KW9975/5, 01KW9976/8, 01KW9977/0 and 01KW0114, the Robert Bosch Foundation, Stuttgart, Deutsches Krebsforschungszentrum (DKFZ), Heidelberg, the Institute for Prevention and Occupational Medicine of the German Social Accident Insurance, Institute of the Ruhr University Bochum (IPA), Bochum, as well as the Department of Internal Medicine, Evangelische Kliniken Bonn gGmbH, Johanniter Krankenhaus, Bonn, Germany. The **HEBCS** was supported by the Helsinki University Central Hospital Research Fund, Academy of Finland (266528), the Finnish Cancer Society, The Nordic Cancer Union and the Sigrid Juselius Foundation. HEBCS would like to thank Drs. Tuomas Heikkinen and Karl von Smitten and RN Irja Erkkilä for their help with the HEBCS data and samples. The population allele and genotype frequencies for controls for the HEBCS GWAS were obtained from the data source funded by the Nordic Center of Excellence in Disease Genetics based on samples regionally selected from Finland, Sweden and Denmark. The **HERPACC** was supported by a Grant-in-Aid for Scientific Research on Priority Areas from the Ministry of Education, Science, Sports, Culture and Technology of Japan, by a Grant-in-Aid for the Third Term Comprehensive 10-Year Strategy for Cancer Control from Ministry Health, Labour and Welfare of Japan, by Health and Labour Sciences Research Grants for Research on Applying Health Technology from Ministry Health, Labour and Welfare of Japan, by National Cancer Center Research and Development Fund and by a grant from Takeda Science Foundation. The **HMBCS** was supported by a grant from the Friends of Hannover Medical School and by the Rudolf Bartling

Foundation. We thank Peter Hillemanns, Hans Christiansen and Johann H. Karstens. **HUBCS** was supported by a grant from the Ministry of Education and Science of the Russian Federation (number 14.574.21.0026, agreement dated June 17, 2014, a unique identifier agreement RFMEFI57414X0026). Financial support for **KARBAC** was provided through the regional agreement on medical training and clinical research (ALF) between Stockholm County Council and Karolinska Institutet, the Swedish Cancer Society, The Gustav V Jubilee foundation and and Bert von Kantzows foundation. The **KBCP** was financially supported by the special Government Funding (EVO) of Kuopio University Hospital grants, Cancer Fund of North Savo, the Finnish Cancer Organizations, and by the strategic funding of the University of Eastern Finland. We thank Eija Myöhänen and Helena Kemiläinen. **kConFab** is supported by a grant from the National Breast Cancer Foundation, and previously by the National Health and Medical Research Council (NHMRC), the Queensland Cancer Fund, the Cancer Councils of New South Wales, Victoria, Tasmania and South Australia, and the Cancer Foundation of Western Australia. GCT is supported by the NHMRC. We wish to thank Heather Thorne, Eveline Niedermayr, all the kConFab research nurses and staff, the heads and staff of the Family Cancer Clinics, and the Clinical Follow Up Study (which has received funding from the NHMRC, the National Breast Cancer Foundation, Cancer Australia, and the National Institute of Health (USA)) for their contributions to this resource, and the many families who contribute to kConFab. Financial support for the **AOCS** was provided by the United States Army Medical Research and Materiel Command [DAMD17-01-1-0729], the Cancer Council of Tasmania and Cancer Foundation of Western Australia and the NHMRC [199600]. We thank the AOCS Management Group (D Bowtell, G Chenevix-Trench, A deFazio, D Gertig, A Green, P Webb) and the ACS Management Group (A Green, P Parsons, N Hayward, P Webb, D Whiteman). **LAABC** is supported by grants (1RB-0287, 3PB-0102, 5PB-0018, 10PB-0098) from the California Breast Cancer Research Program. Incident breast cancer cases were collected by the USC Cancer Surveillance Program (CSP) which is supported under subcontract by the California Department of Health. The CSP is also part of the National Cancer Institute's Division of Cancer Prevention and Control Surveillance, Epidemiology, and End Results Program, under contract number N01CN25403. We thank all the study participants and the entire data collection team, especially Annie Fung and June Yashiki. **pKARMA** study was supported by Märit and Hans Rausings Initiative Against Breast Cancer and Cancer Risk Prediction Center, a Linneus Centre (contract 70867902) financed by the Swedish Research Council. **LMBC** is supported by the 'Stichting tegen Kanker' (232-2008 and 196-2010). Diether Lambrechts is supported by the FWO and the KULPFV/10/016-SymBioSysII. We thank Gilian Peuteman, Dominiek Smeets, Thomas Van Brussel and Kathleen Corthouts. The **MARIE** study was supported by the Deutsche Krebshilfe e.V. [70-2892-BR I, 106332, 108253, 108419], the Hamburg Cancer Society, the German Cancer Research Center and the Federal Ministry of Education and Research (BMBF) Germany [01KH0402]. MARIE would like to thank Alina Vrieling, Sabine Behrens, Ursula Eilber, Muhabbet Celik , Stefan Nickels and Lars Beckmann, Thomas Illig, Kirsten Mittelstraß for their valuable contributions to analysis and generation of the GWAS data , and S. Behrens, U. Eilber, and B. Kaspereit for their excellent technical assistance. **MBCSG** is supported by grants from the Italian Association for Cancer Research (AIRC) and by funds from the Italian citizens who allocated the 5/1000 share of their tax payment in support of the Fondazione IRCCS Istituto Nazionale Tumori, according to Italian laws (INT-Institutional strategic projects "5x1000"). We thank, Paolo Radice, Siranoush Manoukian, Bernard Peissel and Daniela Zaffaroni of the Fondazione IRCCS Istituto Nazionale dei Tumori (INT), Bernardo Bonanni Monica Barile and Irene Feroce of the Istituto Europeo di Oncologia (IEO), and the personnel of the Cogentech Cancer Genetic Test Laboratory. The **MCBCS** was supported by the NIH grants CA116167, CA176785 an NIH Specialized Program of Research Excellence (SPORE) in Breast Cancer [CA116201], and the Breast Cancer Research Foundation and a generous gift from the David F. and Margaret T. Grohne Family Foundation. MCBCS would like to thank Curtis Olswold, Xianshu Wang, and Seth Slettedahl. .. **MCCS** cohort recruitment was funded by VicHealth and Cancer Council Victoria. The MCCS was further supported by Australian NHMRC grants 209057, 251553 and 504711 and by infrastructure provided by Cancer Council Victoria. Cases and their vital status were ascertained through the Victorian Cancer Registry (VCR) and the Australian Institute of Health and Welfare (AIHW), including the National Death Index. The **MEC** was support by NIH grants CA63464, CA54281, CA098758 and CA132839. The work of **MTLGEBCS** was supported by the

Quebec Breast Cancer Foundation, the Canadian Institutes of Health Research for the "CIHR Team in Familial Risks of Breast Cancer" program – grant # CRN-87521 and the Ministry of Economic Development, Innovation and Export Trade – grant # PSR-SIIRI-701. This work was also supported by the Government of Canada through Genome Canada and the Canadian Institutes of Health Research, and the Ministère de l'Économie, Innovation et Exportation du Québec through Genome Québec. We would like to thank Martine Tranchant (CHU de Québec Research Center), Marie-France Valois, Annie Turgeon and Lea Heguy (McGill University Health Center, Royal Victoria Hospital; McGill University) for DNA extraction, sample management and skillful technical assistance. J.S. is Chairholder of the Canada Research Chair in Oncogenetics. **MYBRCA** is funded by research grants from the Malaysian Ministry of Science, Technology and Innovation (MOSTI), Malaysian Ministry of Higher Education (UM.C/HlR/MOHE/06) and Cancer Research Initiatives Foundation (CARIF). Additional controls were recruited by the Singapore Eye Research Institute, which was supported by a grant from the Biomedical Research Council (BMRC08/1/35/19/550), Singapore and the National medical Research Council, Singapore (NMRC/CG/SERI/2010). We thank Phuah Sze Yee, Peter Kang, Kang In Nee, Kavitta Sivanandan, Shivaani Mariapun, Yoon Sook-Yee, Daphne Lee, Teh Yew Ching and Nur Aishah Mohd Taib for DNA Extraction and patient recruitment. The **NBCS** was supported by grants from the Norwegian Research council, 155218/V40, 175240/S10 to ALBD, FUGE-NFR 181600/V11 to VNK and a Swizz Bridge Award to ALBD. NBCS includes the following clinical collaborators: Prof. Anne-Lise Borresen-Dale, Ph.D. (Department of Genetics, Institute for Cancer Research, Oslo University Hospital, Radiumhospitalet, Oslo, Norway and Institute of Clinical Medicine, Faculty of Medicine, University of Oslo (UiO), Oslo, Norway), Prof. Per Eystein Lønning, MD (Section of Oncology, Institute of Medicine, University of Bergen and Department of Oncology, Haukeland University Hospital, Bergen, Norway), Prof. Em. Sophie D. Fosså, MD (National Resource Centre for Long-term Studies after Cancer, Rikshospitalet-Radiumhospitalet Cancer Clinic Montebello, Oslo, Norway), Head physician Tone Ikdahl, MD (Department of Oncology, Oslo University Hospital, Oslo, Norway), Dr. Lars Ottestad, MD (Department of Genetics and Department of Oncology, Oslo University Hospital Radiumhospitalet), Dr. Marit Muri Holmen, MD (Department of Radiology, Oslo University Hospital Radiumhospitalet, Oslo, Norway), Dr. Vilde Haakensen, MD (Department of Genetics and Department of Oncology, Oslo University Hospital Radiumhospitalet and Institute for Clinical Medicine, Faculty of Medicine, University of Oslo, Oslo, Norway), Prof. Bjørn Naume, MD (Division of Cancer Medicine and Radiotherapy, Department of Oncology, Oslo University Hospital Radiumhospitalet, Oslo, Norway), Prof. Eiliv Lund (Department of Community Medicine, Faculty of Health Sciences, University of Tromsø - The Arctic University of Norway, Tromsø, Norway) , Assoc. Prof. Åslaug Helland, MD (Department of Genetics, Institute for Cancer Research and Department of Oncology, Oslo University Hospital Radiumhospitalet, Oslo, Norway and Institute of Clinical Medicine, Faculty of Medicine, University of Oslo, Oslo, Norway), Prof. Inger Torhild Gram, MD (Department of Community Medicine, Faculty of Health Sciences, University of Tromsø and Norwegian Centre for Integrated Care and Telemedicine, University Hospital of North Norway, Tromsø, Norway), Prof. Em. Rolf Kåresen, MD (Department of Breast and Endocrine Surgery, Institute for Clinical Medicine, Ullevaal Hospital, Oslo University Hospital and Institute of Clinical Medicine, Faculty of Medicine, University of Oslo, Oslo, Norway), Dr. Ellen Schlichting, MD (Department for Breast and Endocrine Surgery, Oslo University Hospital Ullevaal, Oslo, Norway), Prof. Toril Sauer, MD (Department of Pathology at Akershus University hospital, Lørenskog, Norway), Dr. Olav Engebråten, MD (Institute for Clinical Medicine, Faculty of Medicine, University of Oslo and Department of Oncology, Oslo University Hospital, Oslo, Norway), Dr. Margit Riis, MD (Department of Surgery, Akershus University Hospital and Department of Clinical Molecular Biology (EpiGen), Institute of Clinical Medicine, Akershus University Hospital, University of Oslo, Lørenskog, Norway). The **NBHS** was supported by NIH grant R01CA100374. Biological sample preparation was conducted the Survey and Biospecimen Shared Resource, which is supported by P30 CA68485. We thank study partcipants and research staff for their contributions and commitment to this study. The **OBCS** was supported by research grants from the Finnish Cancer Foundation, the Academy of Finland (grant number 250083, 122715 and Center of Excellence grant number 251314), the Finnish Cancer Foundation, the Sigrid Juselius Foundation, the University of Oulu, the University of Oulu Support Foundation and the special Governmental EVO funds for Oulu University Hospital-based research activities. We thank

Saila Kauppila, Meeri Otsukka and Kari Mononen. The Ontario Familial Breast Cancer Registry (**OFBCR**) was supported by grant UM1 CA164920 from the National Cancer Institute (USA). The content of this manuscript does not necessarily reflect the views or policies of the National Cancer Institute or any of the collaborating centers in the Breast Cancer Family Registry (BCFR), nor does mention of trade names, commercial products, or organizations imply endorsement by the USA Government or the BCFR. The **ORIGO** study was supported by the Dutch Cancer Society (RUL 1997-1505) and the Biobanking and Biomolecular Resources Research Infrastructure (BBMRI-NL CP16). We thank E. Krol-Warmerdam, and J. Blom for patient accrual, administering questionnaires, and managing clinical information. The LUMC survival data were retrieved from the Leiden hospital-based cancer registry system (ONCDOC) with the help of Dr. J. Molenaar. The **PBCS** was funded by Intramural Research Funds of the National Cancer Institute, Department of Health and Human Services, USA. Louise Brinton, Mark Sherman, Neonila Szeszenia-Dabrowska, Beata Peplonska, Witold Zatonski, Pei Chao, Michael Stagner. The **RBCS** was funded by the Dutch Cancer Society (DDHK 2004-3124, DDHK 2009-4318). RBCS would like to thank Petra Bos, Jannet Blom, Ellen Crepin, Anja Nieuwlaat, Annette Heemskerk and the Erasmus MC Family Cancer Clinic. The **SASBAC** study was supported by funding from the Agency for Science, Technology and Research of Singapore (A*STAR), the US National Institute of Health (NIH) and the Susan G. Komen Breast Cancer Foundation. The **SBCGS** was supported primarily by NIH grants R01CA64277, R01CA148667, and R37CA70867. Biological sample preparation was conducted the Survey and Biospecimen Shared Resource, which is supported by P30 CA68485. The scientific development and funding of this project were, in part, supported by the Genetic Associations and Mechanisms in Oncology (GAME-ON) Network U19 CA148065. We thank study partcipants and research staff for their contributions and commitment to this study. The **SBCS** was supported by Yorkshire Cancer Research S295, S299, S305PA and Sheffield Exeprimental Cancer Medicine Centre. We thank Sue Higham, Helen Cramp, Ian Brock, Sabapathy Balasubramanian and Dan Connley. **SEARCH** was funded by a programme grant from Cancer Research UK [C490/A10124] and supported by the UK National Institute for Health Research Biomedical Research Centre at the University of Cambridge. We thank the SEARCH and EPIC-Norfolk teams for their support. AMD has been supported by Cancer Research UK grant [C8197/A10865] and by the Joseph Mitchell Fund. The **SCCS** is supported by a grant from the National Institutes of Health (R01 CA092447). Data on SCCS cancer cases used in this publication were provided by the Alabama Statewide Cancer Registry; Kentucky Cancer Registry, Lexington, KY; Tennessee Department of Health, Office of Cancer Surveillance; Florida Cancer Data System; North Carolina Central Cancer Registry, North Carolina Division of Public Health; Georgia Comprehensive Cancer Registry; Louisiana Tumor Registry; Mississippi Cancer Registry; South Carolina Central Cancer Registry; Virginia Department of Health, Virginia Cancer Registry; Arkansas Department of Health, Cancer Registry, 4815 W. Markham, Little Rock, AR 72205. The Arkansas Central Cancer Registry is fully funded by a grant from National Program of Cancer Registries, Centers for Disease Control and Prevention (CDC). Data on SCCS cancer cases from Mississippi were collected by the Mississippi Cancer Registry which participates in the National Program of Cancer Registries (NPCR) of the Centers for Disease Control and Prevention (CDC). The contents of this publication are solely the responsibility of the authors and do not necessarily represent the official views of the CDC or the Mississippi Cancer Registry. **SEBCS** was supported by the BRL (Basic Research Laboratory) program through the National Research Foundation of Korea funded by the Ministry of Education, Science and Technology (2012-0000347). **SGBCC** is funded by the National Medical Research Council start-up Grant and Centre Grant (NMRC/CG/NCIS /2010). The study would like to thank the participants and research coordinator Kimberley Chua. Additional controls were recruited by the Singapore Consortium of Cohort Studies-Multi-ethnic cohort (SCCS-MEC), which was funded by the Biomedical Research Council, grant number: 05/1/21/19/425. **SKKDKFZS** is supported by the DKFZ. We thank all study participants, clinicians, family doctors, researchers and technicians for their contributions and commitment to this study. The **SZBCS** was supported by Grant PBZ_KBN_122/P05/2001 and PBZ-KBN-042/P05/2004. The **TBCS** was funded by The National Cancer Institute Thailand. The **TNBCC** was supported by: a Specialized Program of Research Excellence (SPORE) in Breast Cancer (CA116201), a grant from the Breast Cancer Research Foundation, a generous gift from the David F. and Margaret T. Grohne Family Foundation, the Stefanie Spielman Breast Cancer fund and the OSU Comprehensive Cancer Center, the Hellenic

Cooperative Oncology Group research grant (HR R_BG/04) and the Greek General Secretary for Research and Technology (GSRT) Program, Research Excellence II, the European Union (European Social Fund – ESF), and Greek national funds through the Operational Program "Education and Lifelong Learning" of the National Strategic Reference Framework (NSRF) - ARISTEIA. We thank Curtis Olswold, Xianshu Wang, and Seth Slettedahl from Mayo Clinic for genotyping and statistical support for TNBCC. Robert Pilarski and Charles Shapiro were instrumental in the formation of the OSU Breast Cancer Tissue Bank. We thank the Human Genetics Sample Bank for processing of samples and providing OSU Columbus area control samples. The **TWBCS** is supported by the Taiwan Biobank project of the Institute of Biomedical Sciences, Academia Sinica, Taiwan. The **UKBGS** is funded by Breast Cancer Now and the Institute of Cancer Research (ICR), London. ICR acknowledges NHS funding to the NIHR Biomedical Research Centre. We thank Breast Cancer Now and the Institute of Cancer Research for support and funding of the Breakthrough Generations Study, and the study participants, study staff, and the doctors, nurses and other health care providers and health information sources who have contributed to the study. We acknowledge NHS funding to the Royal Marsden/ICR NIHR Biomedical Research Centre.

For Prostate Cancer Association Group to Investigate Cancer Associated Alterations in the Genome (PRACTICAL) studies:

The Department of Medical Epidemiology and Biostatistics, Karolinska Institute, Stockholm, Sweden was supported by the Cancer Risk Prediction Center (CRisP; www.crispcenter.org), a Linneus Centre (Contract ID 70867902) financed by the Swedish Research Council, Swedish Research Council (grant no K2010-70X-20430-04-3), the Swedish Cancer Foundation (grant no 09-0677), the Hedlund Foundation, the Soederberg Foundation, the Enqvist Foundation, ALF funds from the Stockholm County Council. Stiftelsen Johanna Hagstrand och Sigfrid Linner's Minne, Karlsson's Fund for urological and surgical research.
The ESTHER study was supported by a grant from the Baden Württemberg Ministry of Science, Research and Arts. Additional cases were recruited in the context of the VERDI study, which was supported by a grant from the German Cancer Aid (Deutsche Krebshilfe).
The FHCRC studies were supported by grants RO1CA056678, RO1CA082664, and RO1CA092579 from the US National Cancer Institute, National Institutes of Health, with additional support from the Fred Hutchinson Cancer Research Center.
The IPO-Porto study was in part funded by Liga Portuguesa Contra o Cancro.
The Mayo group was supported by the US National Cancer Institute (R01CA72818).
The Prostate Cancer Program of Cancer Council Victoria also acknowledge grant support from The National Health and Medical Research Council, Australia (126402, 209057, 251533, , 396414, 450104, 504700, 504702, 504715, 623204, 940394, 614296,), VicHealth, Cancer Council Victoria, The Prostate Cancer Foundation of Australia, The Whitten Foundation, PricewaterhouseCoopers, and Tattersallï¾’s. EAO, DMK, and EMK acknowledge the Intramural Program of the National Human Genome Research Institute for their support.
The MEC was support by NIH grants CA63464, CA54281 and CA098758.
The Moffitt group was supported by the US National Cancer Institute (R01CA128813, PI: J.Y. Park).
The PCMUS study was supported by the Bulgarian National Science Fund, Ministry of Education and Science (contract DOO-119/2009; DUNK01/2-2009; DFNI-B01/28/2012) with additional support from the Science Fund of Medical University - Sofia (contract 51/2009; 8I/2009; 28/2010).
ProtecT would like to acknowledge finanical support from The University of Cambridge and Cancer Research UK. Cancer Research UK grants [C8197/A10123] and [C8197/A10865] supported the genotyping team. ProtecT was also supported by the National Institute for Health Research (NIHR) which funds the Cambridge Bio-medical Research Centre, Cambridge, UK and the National Cancer Research Prostate Cancer: Mechanisms of Progression and Treatment (PROMPT) collaborative (grant code G0500966/75466) which has funded tissue and urine collections in Cambridge. The NIHR Cambridge Biomedical Research Centre, the DOH HTA (ProtecT grant) and the NCRI / MRC (ProMPT grant) supported the bio-repository. The UK Department of Health funded the ProtecT study through the NIHR Health Technology Assessment Programme (projects 96/20/06, 96/20/99). The ProtecT trial and its linked ProMPT and CAP (Comparison Arm for ProtecT) studies are

supported by Department of Health, England; Cancer Research UK grant number C522/A8649, Medical Research Council of England grant number G0500966, ID 75466 and The NCRI, UK. The epidemiological data for ProtecT were generated though funding from the Southwest National Health Service Research and Development. DNA extraction in ProtecT was supported by USA Dept of Defense award W81XWH-04-1-0280, Yorkshire Cancer Research and Cancer Research UK.
The QLD research is supported by The National Health and Medical Research Council, Australia Project Grant [390130, 1009458] and Enabling Grant [614296 to APCB]; the Prostate Cancer Foundation of Australia (Project Grant [PG7] and Research infrastructure grant [to APCB]).
SCCS is funded by NIH grant R01 CA092447, and SCCS sample preparation was conducted at the Epidemiology Biospecimen Core Lab that is supported in part by the Vanderbilt-Ingram Cancer Center (P30 CA68485).
SEARCH is funded by a programme grant from Cancer Research UK [C490/A10124] and supported by the UK National Institute for Health Research Biomedical Research Centre at the University of Cambridge.
The Tampere (Finland) study was supported by the Academy of Finland (251074), The Finnish Cancer Organisations, Sigrid Juselius Foundation, and the Competitive Research Funding of the Tampere University Hospital (9N069 and X51003).
UKGPCS would also like to thank the following for funding support: The Institute of Cancer Research and The Everyman Campaign, The Prostate Cancer Research Foundation, Prostate Research Campaign UK (now Prostate Action), The Orchid Cancer Appeal, The National Cancer Research Network UK, The National Cancer Research Institute (NCRI) UK, and NIHR funding to the NIHR Biomedical Research Centre at The Institute of Cancer Research and The Royal Marsden NHS Foundation Trust.
The Ulm group received funds from the German Cancer Aid (Deutsche Krebshilfe).
The Keith and Susan Warshaw Fund, C. S. Watkins Urologic Cancer Fund and The Tennity Family Fund supported the Utah study. The project was supported by Award Number P30CA042014 from the National Cancer Institute.

For Ovarian Cancer Association Consortium (OCAC) studies:

Funding of the constituent studies was provided by the American Cancer Society (CRTG-00-196-01-CCE); the California Cancer Research Program (00-01389V-20170, N01-CN25403, 2II0200); the Canadian Institutes for Health Research (MOP-86727); Cancer Council Victoria; Cancer Council Queensland; Cancer Council New South Wales; Cancer Council South Australia; Cancer Council Tasmania; Cancer Foundation of Western Australia; the Cancer Institute of New Jersey; the Celma Mastry Ovarian Cancer Foundation ; the Danish Cancer Society (94-222-52); the ELAN Program of the University of Erlangen-Nuremberg; the Eve Appeal; the Helsinki University Central Hospital Research Fund; Helse Vest; Imperial Experimental Cancer Research Centre (C1312/A15589); the Norwegian Cancer Society; the Norwegian Research Council; Nationaal Kankerplan of Belgium; Grant-in-Aid for the Third Term Comprehensive 10-Year Strategy for Cancer Control from the Ministry of Health Labour and Welfare of Japan; the L & S Milken Foundation; the Polish Ministry of Science and Higher Education (4 PO5C 028 14, 2 PO5A 068 27); Malaysian Ministry of Higher Education (UM.C/HlR/MOHE/06) and Cancer Research Initiatives Foundation; the Roswell Park Cancer Institute Alliance Foundation; the US National Cancer Institute (K07-CA095666, K07-CA143047, K22-CA138563, N01-CN55424, N01-PC067010, N01-PC035137, P01-CA017054, P01-CA087696, P30-CA15083, P50-CA105009, P50- CA136393, R01-CA014089, R01-CA016056, R01-CA017054, R01-CA049449, R01-CA050385, R01-CA054419, R01- CA058598, R01-CA058860, R01-CA061107, R01-CA061132, R01-CA063682, R01-CA064277, R01-CA067262, R01-CA071766, R01-CA074850, R01-CA076016, R01-CA080742, R01-CA080978, R01-CA083918, R01-CA087538, R01- CA092044, R01-095023, R01-CA106414, R01-CA122443, R01-CA112523, R01-CA114343, R01-CA126841, R01- CA136924, R01-CA149429, R03-CA113148, R03-CA115195, R37-CA070867, R37-CA70867, U01-CA069417, U01- CA071966, K07-CA80668, P50-CA159981, R01CA095023, MO1- RR000056, R01-CA063678, UM1-CA186107, P01-CA87969, R01-CA49449, UM1-CA176726, R01-CA67262, and Intramural research funds); the US Army Medical Research and Material Command (DAMD17-98-1- 8659, DAMD17-01-1-0729, DAMD17-

02-1-0666, DAMD17-02-1-0669, W81XWH-10-1-0280); the National Health and Medical Research Council of Australia (199600 and 400281); the German Federal Ministry of Education and Research of Germany Programme of Clinical Biomedical Research (01 GB 9401); the state of Baden-Württemberg through Medical Faculty of the University of Ulm (P.685); the Minnesota Ovarian Cancer Alliance; the Mayo Foundation; the Fred C. and Katherine B. Andersen Foundation; the Lon V. Smith Foundation (LVS-39420); the Oak Foundation; the OHSU Foundation; the Mermaid I project; the Rudolf-Bartling Foundation; the UK National Institute for Health Research Biomedical Research Centres at the University of Cambridge, Imperial College London, the Royal Marsden Hospital; WorkSafeBC.
